# Silicone Breast Implants as a Model System for Understanding Polymer Permeation in Soft Materials In Vivo

**DOI:** 10.1002/adhm.71641

**Published:** 2026-08-24

**Authors:** D. Bouyer, H. Mutlu, C. Charmette, E. Randrianaridera, Z. Adaidi, R. Hedna, L. Pieuchot, I. Pluvy, K. Anselme, N. Couturier, J. P. Mericq, A. Ponche, I. Brigaud

**Affiliations:** ^1^ Institut Européen des Membranes CNRS IEM Université De Montpellier Montpellier France; ^2^ Department of Chemistry Technical Polymer Chemistry Rheinland‐Pfälzische Technische Universität Kaiserslautern‐Landau (RPTU) Erwin‐Schrödinger‐Straße Kaiserslautern Germany; ^3^ Institut de Science des Matériaux de Mulhouse UMR 7361 CNRS/Université De Haute Alsace Mulhouse Cedex France; ^4^ Laboratory of Organic and Macromolecular Chemistry (IOMC) Friedrich Schiller University Jena Humboldtstr. 10 Germany; ^5^ Helmholtz Institute for Polymers in Energy Applications Jena (HIPOLE Jena) Jena Germany; ^6^ Helmholtz‐Zentrum Berlin for Materials and Energy GmbH Berlin Germany; ^7^ Leibniz‐Institut für Verbundwerkstoffe (IVW) GmbH Erwin‐Schrödinger‐Straße 58 Kaiserslautern Germany; ^8^ CHU Besançon Laboratoire SINERGIES Service D'orthopédie Traumatologie et Chirurgie Plastique Université De Franche‐Comté Besançon France

**Keywords:** biointerface, gel bleed, polymer permeation, silicone breast implant, solution–diffusion

## Abstract

Silicone breast implants have long been associated with the gradual release of low molecular weight siloxanes from the inner gel through the implant membrane into the periprosthetic biological environment, a phenomenon commonly referred to as “gel bleed.” Despite more than five decades of investigation spanning polymer science, transport theory, and biomedical research, the physicochemical mechanisms underlying silicone permeation remain conceptually fragmented. Here, we revisit silicone permeation by examining how molecular transport arises from the intrinsic interplay between polymer architecture, thermodynamic partitioning, and diffusional mobility within crosslinked networks. We further analyze the key determinants of permeation, including implant aging, barrier‐layer technologies, and interaction with the periprosthetic tissue environment. We further discuss how dynamic bio‐interfaces influence bidirectional transport and bioaccumulation in vivo. Beyond their implications for implant performance, these observations provide broader conceptual insights into mass transport in soft materials, highlighting general principles relevant to elastomers, hydrogels, and drug‐delivery systems. By integrating material structure, transport physics and host interactions, this review establishes a mechanistic viewpoint on polymer permeation in vivo and offers design considerations for next‐generation soft implantable biomaterials.

## Graphical abstract

Silicone breast implants are presented as a model system for understanding polymer permeation in vivo. Rather than representing material failure, “gel bleed” emerges from solution–diffusion transport‐mediated. By integrating polymer architecture, physicochemical transport, and biointerfacial processes, this review provides a unified framework that reconciles decades of observations on silicone implant.

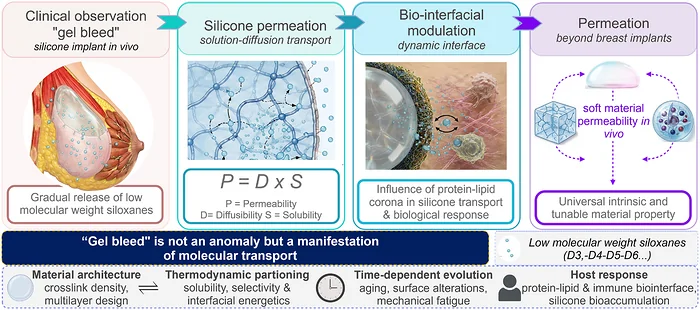

## 1 Introduction

Soft polymeric biomaterials used in vivo are dynamic systems whose long‐term performance is governed not only by their mechanical properties but also by thermodynamic and kinetic processes controlling molecular transport across polymer networks and biological interfaces. In dense polymer systems, these processes are commonly described by solution–diffusion theory, where permeability emerges from the coupled effects of penetrant solubility and diffusivity within the material. Despite its broad relevance in polymer science, this framework has rarely been systematically applied to implantable biomaterials, where discussions of long‐term performance often emphasize mechanical failure, structural degradation, and host response while often underestimating the role of molecular transport. This disconnect is particularly evident in silicone breast implants, one of the most extensively studied soft implantable systems. The introduction of silicone gel‐filled implants in the early 1960’ revolutionized the medical implant industry. Among them, breast implants have enjoyed a meteoric rise in popularity, dominating the aesthetic, restorative, and reconstructive surgery market from their first implantation in 1962 to the present day [1, 2]. Their widespread clinical use, long implantation times, and the substantial body of clinical, experimental, and materials‐science literature have made them a unique model system for examining molecular transport in soft materials under physiological conditions.

Along six generations of innovation, the basic structure of breast implants has remained largely the same: a solid, cross‐linked elastomer membrane filled with silicone gel. Silicone's popularity is primarily due to its mechanical properties, chemical stability, and presumed biocompatibility. At the molecular level, advantageous material properties derive from the physicochemical characteristics of polydimethylsiloxane (PDMS), a silicon‐based polymer with an exceptionally flexible Si–O–Si backbone, chemical stability, optical transparency, tunable mechanical properties, low glass transition temperature (−120°C), high segmental mobility, and pronounced hydrophobicity due to its low surface energy [3, 4, 5, 6, 7]. Together, these features confer softness, elasticity, and long‐term mechanical resilience, while also enabling the material to withstand substantial deformation with low rupture rates. Beyond its clinical relevance, the silicone breast implant can be viewed as a model system for studying molecular transport in soft polymeric materials under physiological conditions. More broadly, it represents a class of dynamic, multicomponent systems in which polymer architecture, penetrant chemistry, and biological interfaces are intrinsically coupled. This physicochemical framework provides the basis for understanding long‐term molecular transport across implantable polymeric materials.

However, another reality challenges this idealized perception. These same properties inherently favor the continuous transfer of low molecular weight siloxanes (LMWS) from the inner gel through the implant membrane into the periprosthetic environment. From a materials‐science perspective, implant's architecture constitutes a coupled polymer system in which a dense crosslinked PDMS elastomer encloses a gel phase that itself contains both a crosslinked network and a mobile extractable fraction. This design creates the conditions for long‐term molecular exchange. Mobile LMWS remain thermodynamically capable of partitioning into the surrounding elastomer membrane and subsequently diffusing into the periprosthetic environment. Accordingly, the phenomenon commonly referred to as “gel bleed” can be interpreted within the established solution–diffusion framework governing mass transport in dense polymer systems. Throughout this review, this framework serves as the physicochemical basis for discussing silicone transport across intact implant membranes.

Finally, silicone products released from silicone implants expose the patient to health risks and undermine implant safety and longevity. As early as 1977, silicone permeation could be observed macroscopically [8, 9]. Surgeons called this phenomenon “silicone bleeding” [10]. Implant membranes were permeable to silicone gel [9], thereby facilitating the diffusion of potential immunoreactive contaminants into the periprosthetic tissue among which LMWS [11]. Accordingly, implant rupture is considered the extreme case of gel permeation [12]. Since then, manufacturers have sought to mitigate permeation by refining the physicochemical properties of implants. Design improvements have focused on membrane reinforcement, enhanced barrier function, and increased gel cohesiveness. In the late 1990’, manufacturers added texturation to the outer surface of breast implant to promote better anchoring of the breast tissue to the implant which is particularly important for breast reconstruction. Over time, solid fragments have been shown to detach from the silicone membrane due to wear and fatigue [13, 14, 15], cumulating with silicone permeation phenomenon. This distinction is crucial, because two mechanistically different forms of silicone exposure may coexist around implants: first, molecular permeation through an intact membrane; and second, release of particulate or solid debris generated by mechanical damage, abrasion, or surface erosion. These forms of release likely differ in their kinetics, spatial distribution, cellular uptake, and immunological consequences, yet they are often conflated in the literature under a shared vocabulary of “silicone bleeding”. In 2019, surface texturation has been correlated to the onset of a rare cancer called Breast Implant‐Associated Anaplastic Large Cell Lymphoma (BIA‐ALCL) [16] and textured implants were withdrawn from the market. Consequently, research over the last two decades has increasingly focused on the biological consequences of surface topography and particulate debris release [15, 17]. During the same period of time, gel permeation, despite representing an intrinsic feature of silicone‐based implant systems, received comparatively less attention.

Periprosthetic tissues are the primary recipients of silicone species released from the implant. Silicone device implantation triggers an immediate immune reaction known as the foreign body response (FBR) which starts with the deposition of plasma proteins and lipids on the material and culminates in the formation of a periprosthetic tissue that surrounds and isolates the implant from the host body [18, 19]. Experimental evidence suggests that permeating silicone species accumulate in the periprosthetic tissues and locally reactivate the FBR, favoring a chronic immune reaction environment [20]. In addition, silicone residues have been located beyond the periprosthetic tissue, in intramammary lymphatic nodes and other organs [12, 21, 22, 23], where they may trigger systemic chronic inflammation, even following implant removal [24]. These observations indicate that long‐term implant performance is influenced not only by the bulk properties of the material but also by interactions occurring at the interface between polymer transport and host biology. It is instead shaped at the interface between polymer physics and host biology, where the release, partitioning, accumulation, and transformation of silicone species may influence plasmatic molecule deposition and maturation, the persistence of inflammatory signaling, and potentially the dissemination of material beyond the implantation site.

Despite extensive technical advances, silicone permeation has been observed in all generations of implants and remains a contemporary challenge in breast implant manufacturing. The FDA recognizes gel permeation as an intrinsic feature of all silicone implants, regardless of membrane design or gel cohesiveness, and recommends comprehensive evaluations to characterize this phenomenon and provide transparent information to patients [25]. This regulatory position is consistent with the understanding that silicone permeation is an intrinsic transport phenomenon whose extent depends on material composition and barrier properties. In that, even highly cohesive gels and multilayer membranes cannot be assumed to constitute impermeable systems when the penetrant and barrier share strong chemical affinity.

Curiously, although silicone permeation has been acknowledged for over 50 years, understanding of the process remains limited. This knowledge gap likely reflects its interdisciplinary nature, spanning polymer chemistry, the physics of permeation, and biology. It also reflects a persistent conceptual divide between clinical descriptions of silicone implant “bleed” and the formal frameworks used in membrane science to describe transport in dense polymers. In this context, this review refocuses attention on this long‐standing issue and reexamines it. It aims to clarify the terminology used, provide an updated description of the chemical composition of both the silicone gel and implant membrane, explain the principles of permeation, and explore how silicone compounds interact with the host, particularly through the role of plasma proteins and lipids deposited at the implant interface. This review does not address the potential associations between silicone exposure and silicone‐related diseases, as these have been comprehensively discussed elsewhere [26, 27, 28, 29, 30]. This review critically examines the evolution of silicone breast implants through the lens of polymer transport and discusses persistent silicone permeation within the established solution–diffusion framework. It integrates current knowledge on implant composition, transport mechanisms, and biological interactions, highlighting silicone breast implants as a clinically relevant model for understanding molecular transport in soft biomaterials. Finally, it identifies current knowledge gaps and outlines future research directions to advance the understanding of silicone transport in vivo.

## 2 Silicone Implant Evolution as Evidence of Persistent Molecular Transport

The evolution of silicone breast implants provides a unique longitudinal model for examining how successive material redesigns have sought to address persistent challenges of mechanical reliability, biocompatibility, and molecular containment (Figure 1). Across six generations, manufacturers introduced iterative modifications to shell architecture, gel cohesiveness, and surface engineering in response to recurring clinical complications and safety concerns. Viewed through the lens of polymer transport, these technological developments also influenced in the transport properties of the implant shell by modifying membrane architecture, gel cohesiveness, and barrier characteristics. Importantly, while these developments substantially improved device performance, none eliminated the underlying phenomenon of silicone permeation. The first breast implants were developed in the late 19th century from raw materials as diverse as ivory, glass, ox cartilage, ground rubber, and polyester. In 1961, plastic surgeons Frank Gerow and Thomas Cronin collaborated with Dow Corning Corporation, a company specializing in silicone chemistry, to develop the first silicone breast implant. Inspired by the shape and consistency of a blood bag, this innovation marked the beginning of six generations of silicone implant design (Figure 1).

**FIGURE 1:**
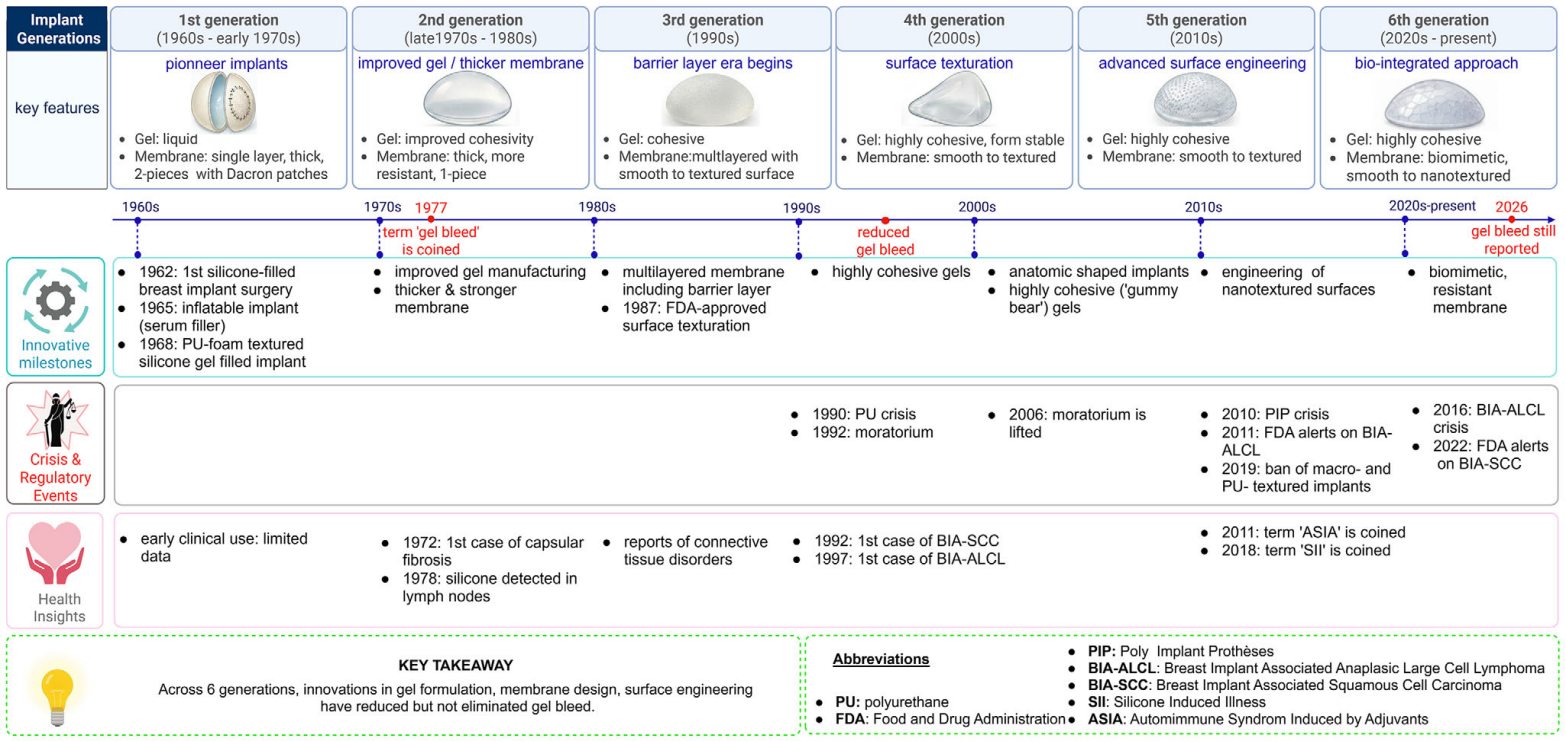
Timeline of silicone breast implant generations: emphasis on technical innovations, health insights and crisis events.

The first generation featured a two‐piece, seam‐joined membrane that was relatively thick (∼0.75 mm) and filled with a liquid viscous PDMS gel with low crosslinking density. The implant surface was smooth and devoid of topography. Posteriorly, patches made of Dacron a synthetic polyester surgical material used to reinforce breast implants–were incorporated to help anchor the implant in place [31, 32]. From a polymer standpoint, early implant designs illustrate the balance between mechanical performance and molecular transport. Lower gel crosslink density improved softness, whereas membrane thickness primarily influenced resistance to molecular diffusion. However, implant rupture and capsular fibrosis, first described in 1972 [33] and later termed *capsular contracture* in 1980 [34], emerged as major complications, prompting the development of the *second generation*. Meanwhile, the implant catalogue expanded to include two alternative models: inflatable saline‐filled implants (1965) and polyurethane (PU) foam‐covered gel implants (1968) [35].

Introduced in 1972, the second generation was characterized by a one‐piece, thicker membrane (∼1.0–1.5 mm) without Dacron patches and contained a less cohesive, more liquid silicone gel intended to create a softer feel. However, cases of fibrosis and high rupture rates continued to be reported and implant membranes were shown to be permeable to silicone gel [31]. The concept of silicone permeation was introduced in 1976 when Barker et al. demonstrated the spontaneous diffusion of silicone materials from the silicone gel filler through the membrane of the implant onto filter paper [9]. In 1977, the term silicone bleeding was coined to describe this phenomenon and is still used in the medical community today [8, 36].

The third generation developed between 1980 and 1990, focused on strengthening the membrane and mitigating gel filler permeation. It marked the introduction of multilayered membrane (0.28–0.3 mm), including a chemically modified fluorosilicone constituting the “barrier layer” [37]. The introduction of multilayer shells incorporating fluorosilicone barrier layers represented an important materials engineering strategy to improve the barrier properties of the implant shell and reduce silicone permeation. This new design significantly reduced the rate of membrane failure. However, this decade is also marked by the emergence of reports linking silicone‐based devices to connective tissue disorders (e.g., scleroderma, rheumatoid arthritis [38, 39, 40]). The substantially lower capsular contracture profile associated with the use of PU coating [41] prompted manufacturers to texture the outer surface of their implants. Paradoxically, PU‐coated implants were withdrawn in 1991 from the US market (though not in Europe) after in vitro studies revealed that PU foam released a toxic and potentially carcinogenic degradation product [42, 43].

The first textured implant approved by the FDA in 1987, marked the advent of the *fourth generation*. Manufacturers adopted various texturing techniques, such as salt loss and imprint stamping, to increase surface roughness and facilitate tissue integration. Most implants were round and filled with a moderately cohesive gel [31]. In the meantime, the early 1990s were marked by growing controversy over implant safety. Despite technical improvements, reports of adverse effects continued to accumulate in the scientific literature, leading to global litigation as patients claimed associations with immune disorders and cancer. In 1992, the first case of breast implant‐associated squamous cell carcinoma (BIA‐SCC) a rare, aggressive, epithelial‐based tumor was reported in the literature [44]. That same year, the US FDA imposed a moratorium on the risks associated with silicone‐filled breast implants, suspending their use in surgery until it ended in 2006 [45].

After the moratorium, the fifth generation emerged, characterized by highly cohesive (“gummy bear”) form‐stable implants. In 2010, the Poly Implant Prothèse (PIP) scandal reignited public concern when it was revealed that industrial‐grade silicone had been used, resulting in an abnormally high rate of ruptures and complications [46]. Lately, the breast implant associated anaplastic large cell lymphoma (BIA‐ALCL) crisis rocked the fourth and fifth generation of breast implants. First reported in 1997 [47], BIA‐ALCL became the subject of an FDA alert in 2011 [48], and was recognized as a distinct pathology in 2016 [49]. As its occurrence has been correlated to macrotextured implant surfaces [50], both PU and textured silicone implants were banned from use in surgery in 2019.

These developments led to the *sixth generation* of implants, featuring biomimetic surfaces, more resistant membranes, and variations in gel consistency. In September 2022, the FDA once again raised awareness of BIA‐SCC, 30 years after the first reported case [51]. In summary, the evolution of breast implants has been driven by successive technological advances aimed at improving implant performance, including enhanced mechanical durability, reduced rupture rate, improved biocompatibility, optimized tissue integration, and reduced silicone permeation. Despite these developments, silicone permeation has remained detectable across successive implant generations (Figure 1). Viewed across six generations, the historical evolution of silicone breast implants illustrates that improvements in implant design have progressively reduced, but not completely eliminated, silicone permeation, highlighting the importance of understanding molecular transport alongside mechanical performance.

## 3 From “Gel Bleed” to Polymer Permeation: Terminology, Misconceptions and Polymer Transport Framework

The literature employs heterogenous and often inconsistent terminology to describe the implant membrane, periprosthetic tissue, and silicone permeation, often leading to conceptual ambiguity. Accordingly, it is essential to first clarify the terminology associated with breast implants.

The *implant membrane* was initially termed as “silicone bag” [8]. Then other terms were used such as (elastomer) shell [52, 53, 54, 55] or envelope [11, 56]. The *tissue formed around the implant* is referred to as periprosthetic tissue [20], periprosthetic capsule [57] capsular tissue [58] or capsule [59, 60, 61], although capsule may also refer to pathological fibrotic periprosthetic tissue. Since silicone breast implants were introduced, silicone permeation has been described by various names as well. In general, the terms used misrepresent the principles underlying this physical phenomenon. The term “gel bleed(ing)” was the first and is still the most commonly used in the literature [8]. Other terms have also been employed, including “silicone leak(age)” [9, 62, 63, 64], “silicone sweat(ing)” [62], “silicone perspiration” [65], “silicone diffusion” [66], “silicone leach” [50] These terms largely describe the observed phenomenon but do not necessarily distinguish between different underlying transport mechanisms. In other words, these terms are largely phenomenological and may lead to a misinterpretation of the underlying material mechanism.

Throughout this review, we therefore use the term silicone permeation to describe the diffusion‐driven transport of low‐molecular‐weight silicone species through an intact implant membrane according to the established solution–diffusion framework. This terminology places the phenomenon within the established framework of mass transport in dense polymer systems and facilitates a mechanistic interpretation of molecular transport across intact implant membranes. The process occurring in intact silicone implants is more rigorously described by the language of polymer transport: the partitioning of mobile siloxane species into a dense elastomer, their diffusion through a non‐porous but highly permeable network, and their desorption into adjacent tissue. Unlike the term “leakage,” which may imply membrane rupture or manufacturing defects, “permeation” describes an intrinsic transport process occurring through an intact polymer network. Likewise, the broad term “silicone” collapses chemically distinct entities into a single category, despite the fact that crosslinked elastomer fragments, linear oligomeric chains, cyclic volatile siloxanes and gel‐associated low‐molecular‐weight species likely differ substantially in mobility, tissue partitioning and biological activity.

From a polymer‐science perspective, the term silicone permeation istherefore, preferred because it aligns clinical observation with physicochemical reality. It distinguishes transport through an intact polymer network from failure‐related events such as rupture, tearing, or release of particulate debris. It also recognizes that the breast implant is not a static object but a multicomponent material system in which the gel, membrane and surrounding tissue form a coupled transport environment (Table 1).

**TABLE 1:** Mechanisms of silicone transport and release: surface debris versus molecular permeation.

Feature	Surface/particulate debris	Molecular permeation
Underlying mechanism	Mechanical damage, wear, abrasion, and surface topography erosion	Intrinsic thermodynamic transport (solution–diffusion) driven by chemical gradients
Implant membrane	Damaged, eroded, or fatigued(micro‐ or macroscopic failure)	Intact
Silicone physical state and size	Solid, particulate fragments of crosslinked elastomer	Solute, mobile species (liquid/volatile) at the molecular or nanometric scale
Chemical composition	Crosslinked PDMS	LMWS including volatile cyclic species (e.g., D4, D5, D6) and linear oligomers
Kinetics of release	Episodic or progressive, linked to physical activity, friction, or device aging	Continuous, sustained long‐term flux determined by molecular mobility and free‐volume physics
Standard histology (H&E) limit	Hard to chemically distinguish from permeation products	hard to chemically distinguish from solid debris

Finally, the term “silicone” encompasses chemically and physically distinct species, including crosslinked elastomer fragments, linear oligomers, and cyclic volatile siloxanes (e.g., D4, D5, D6), which differ markedly in mobility, partitioning behavior, and biological activity. These transport principles are discussed in detail in the following sections. *To ensure clarity in the subsequent sections of this manuscript, the terms “membrane,” “periprosthetic tissue,” and “silicone permeation” will be used consistently*.

## 4 Polymer Transport in Medical Silicone Devices: Breast Implants as a Model System

### 4.1 Physicochemical Mechanisms Underlying Silicone Permeation

#### 4.1.1 Solution–Diffusion Mechanisms in PDMS Elastomer

A silicone breast implant can be conceptualized as a three‐phase system: (i) the silicone gel core which contains the mobile LMWS (Glossary Box.1); (ii) the PDMS (Glossary Box.1) polymer membrane which acts as a dense transport barrier; and (iii) the periprosthetic environment (fluids and tissues) which initially contains negligible concentrations of siloxane (Figure 2A,B). Permeation therefore depends on the coupled properties of the donor phase (the gel), the barrier phase (the membrane), and the receiver phase (the periprosthetic environment), with transport driven by concentration and chemical‐potential gradients between these compartments. Transport of LMWS across a PDMS dense membrane follows the solution–diffusion model, which constitutes the standard theoretical framework for describing mass transfer through dense, non‐porous polymeric materials. In this model, permeation occurs via sequential sorption, diffusion, and desorption steps, and the permeability coefficient (*P*) is defined as: *P = D* × *S* where *D* is the molecular diffusion coefficient (a kinetic parameter describing molecular mobility within the polymer matrix) and *S* is the solubility or partition coefficient (a thermodynamic parameter describing the affinity of the penetrant for PDMS relative to the adjacent phase) (Figure 2B) [67]. Together, these parameters determine the overall permeation rate. The solution–diffusion framework is not specific to silicone elastomers but applies broadly to a wide range of soft materials, including hydrogels, elastomeric membranes, and polymer‐based drug delivery systems. In all these systems, permeability emerges from the coupled contributions of thermodynamic affinity (solubility) and molecular mobility (diffusivity), both of which are governed by polymer structure and environmental interactions. Silicone breast implants therefore represent one clinically relevant application of these established transport principles. In other words, this framework is especially applicable for PDMS because the material is non‐glassy, non‐porous, and highly deformable under physiological conditions. Unlike porous membranes, where transport occurs through fixed channels, penetrants in PDMS move through transient intermolecular voids generated by thermal motion of the polymer chains [68, 69].

**FIGURE 2:**
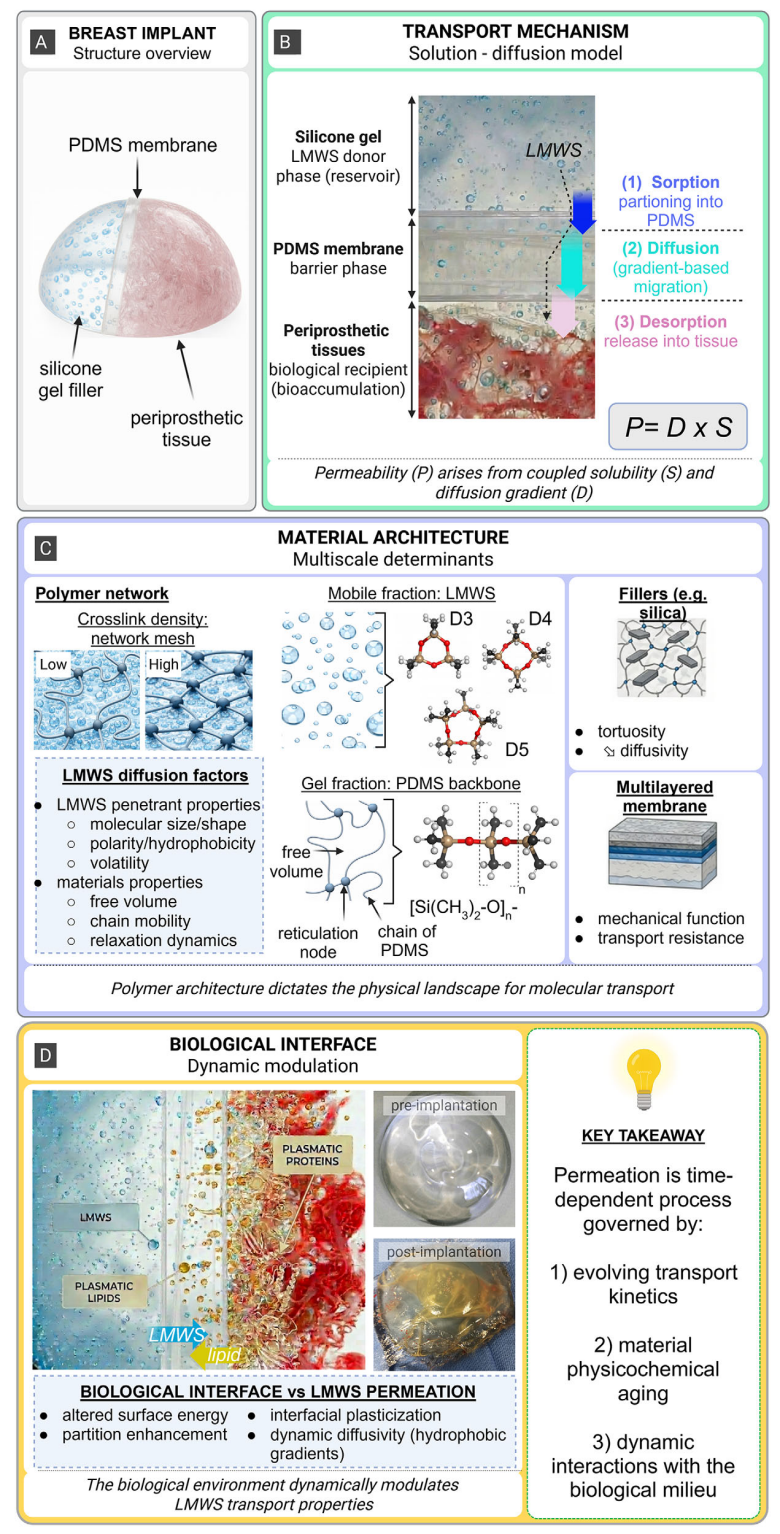
Multiscale determinants of silicone gel permeation in breast implant. (A) Schematic representation of breast implant, (B) three‐steps permeation of LMWS across PDMS membrane, (C) implant chemistry, and (D) biologic interface influences (implant photos from personal archives).

The PDMS composing the membrane is a highly flexible, rubbery polymer with a glass transition temperature, *T*_g_, ≈ −120°C. At physiological temperature (far above *T*_g_), the polymer chains are highly mobile. This mobility generates transient free volume elements within the matrix, permitting small, nonpolar molecules such as LMWS to dissolve and diffuse through the membrane (Figure 2B). At the molecular scale, the unusually low torsional barrier of the Si–O backbone enables rapid segmental rearrangements of PDMS chains, dynamically opening and closing nanoscopic free‐volume cavities. These transient cavities do not constitute permanent pores, but they nevertheless provide sufficient configurational space for small siloxane molecules to undergo stepwise diffusive motion through the network. This molecular mobility contributes to the comparatively high permeability of PDMS toward small hydrophobic penetrants [70, 71]. Experimental studies using lightly crosslinked PDMS membranes have validated this transport mechanism for cyclic siloxanes.

Mechanistically, LMWS migration across the intact membrane proceeds in three sequential steps [72]: (1) *Partitioning* from the gel into the membrane (Glossary Box.2): only the mobile fraction of LMWS in the gel can dissolve into the PDMS membrane to reach the thermodynamic equilibrium between the two phases in contact. This sorption step determines the amount of siloxane entering the membrane. (2) *Fickian diffusion* across the membrane (Glossary Box.2): once sorbed, molecules migrate down their concentration gradient according to Fick's laws. This step is governed by the diffusion coefficient *D* and determines the rate of transport through the polymer. (3) *Desorption into periprosthetic tissue*: upon reaching the outer surface, siloxanes partition into the surrounding biological environment. While molecular diffusion controls intramembrane transport, the interfacial partitioning step determines how efficiently siloxanes exit the implant and enter adjacent tissue (Figure 2B). Gel composition, membrane aging, lipid adsorption, protein deposition and tissue remodeling may all alter either the diffusion coefficient *D* and/or the solubility *S*, or both, thereby changing overall permeability even in the absence of visible structural damage, as discussed in the following sections. These processes provide a physicochemical basis for the slow release of LMWS through intact implant membranes. The structural similarity between LMWS and PDMS favors partitioning into the membrane, whereas transfer into the predominantly aqueous biological environment may impose an additional interfacial resistance. Accordingly, molecular permeation through an intact membrane should be distinguished from release associated with macroscopic rupture or particulate damage.

**Box.1: Silicone chemistry glossary**

**Polydimethylsiloxane (PDMS)**: synthetic silicon‐based polymer composed of repeating –Si(CH_3_)_2_–O– units, typically forming linear or crosslinked networks with high molecular weight; widely used in biomedical devices due to its chemical stability, hydrophobicity, and viscoelastic properties.

**Low‐molecular‐weight siloxanes (LMWS)**: siloxane species with relatively short chain lengths (typically ≤20 siloxane units), encompassing both linear and cyclic structures; they may occur as residual or extractable fractions in silicone materials and generally exhibit higher mobility and bioavailability than crosslinked polymeric forms.

**Cyclic volatile methylsiloxanes (cVMS)**: a subset of LMWS characterized by sufficient vapor pressure to evaporate under ambient conditions, commonly including cyclic species such as D4, D5, and D6; their volatility contributes to facilitated environmental dispersion.

**Linear PDMS oil** refers to uncrosslinked, linear PDMS with low to moderate molecular weight, typically used as a silicone fluid or oil.

#### 4.1.2 Molecular Architecture and Transport Selectivity

Siloxane transport through PDMS membranes is strongly dependent on molecular architecture. In permeation science, molecular architecture influences both kinetic accessibility to free volume and thermodynamic compatibility with the host polymer. For siloxanes, these two dimensions are tightly coupled because structural similarity to PDMS promotes sorption, while molecular size and topology govern the ease of diffusive motion. Transport selectivity arises primarily from differences in molecular size, geometry, and polymer affinity which differentially influence diffusivity (*D*) and solubility (*S*). Because crosslinking is intrinsically incomplete, the gels contain approximately 1%–2% LMWS (3–20 siloxane units), present as both linear and cyclic species [73]. Cyclic volatile methylsiloxanes (D4–D6), exhibit structural features that may favor permeation, including small hydrodynamic radii, compact cyclic geometry, and relatively high volatility (Figure 2C). These characteristics enhance molecular mobility within the PDMS network compared with longer linear siloxanes or higher‐molecular weight PDMS fluids. Experimental studies in lightly crosslinked silicone elastomers confirm this architectural selectivity: D4 diffuses substantially faster than low‐viscosity linear PDMS oils, even when thermodynamic solubility is similar [72]. This observation indicates that molecular geometry predominantly modulates diffusivity (*D*) rather than solubility (*S*). More broadly, solubility reflects chemical affinity, whereas diffusivity reflects the ability of a penetrant to move through transient free volume. Molecules may therefore exhibit comparable partitioning into PDMS but different permeation rates because of differences in size, flexibility, and geometry. From a thermodynamic perspective, siloxanes exhibit strong affinity for PDMS based on chemical similarity. Both share a Si–O–Si backbone, comparable polarity, and similar intermolecular interactions. Consistent with the “like dissolves like” principle (Glossary Box.2), the structural and chemical compatibility between LMWS from the gel and the PDMS membrane may also be rationalized using solubility theory, according which penetrants with cohesive energy densities close to that of PDMS display favorable sorption into the silicone phase. Thus, while geometry primarily governs diffusion kinetics, chemical similarity governs equilibrium sorption. Together, these factors enable LMWS to dissolve into and migrate across intact PDMS membranes. Evidence for the presence of cyclic siloxanes outside the implant has been reported, including gas chromatography‐mass spectrometry (GC‐MS) detection of cyclic siloxanes D4–D6 in the blood and periprosthetic tissues of women with silicone implants [74, 75, 76] (see section “When silicone meets biology”). However, rigorously controlled in vivo studies directly comparing cyclic and linear siloxanes under clinically relevant implantation conditions remain limited. Consequently, current understanding of transport selectivity is derived largely from in vitro diffusion experiments and physicochemical modeling.

**Box.2: Permeation Glossary**

**Partitioning**: the thermodynamic distribution of a solute (such as low molecular weight siloxanes) between two adjacent phases at equilibrium, the donor phase (e.g., the gel) and the recipient (e.g., the PDMS membrane interface).

**Fickian diffusion**: molecular transport driven by a concentration gradient, for which the diffusive flux is proportional to the that gradient.

**“Like dissolves like” principle**: compounds preferentially dissolve in phases with similar chemical polarity and intermolecular interactions. Because both LMWS and the PDMS membrane are non‐polar and chemically similar, LMWS have a favorable affinity for the membrane. In contrast, their lower solubility in aqueous biological fluids may retard, but not necessarily prevent, outward transfer.

#### 4.1.3 Quantifying Silicone Permeation: Diffusion Coefficients and Free Volume Theory

Quantitative diffusion measurements provide insight into the intrinsic permeability of PDMS membrane. For D4 in lightly crosslinked PDMS elastomers, reported diffusion coefficients typically fall in the range of *D* ≈10^−^^1^ ^1^ to 10^−^^1^ ^2^ m^2^ s^−^^1^, exhibiting classical Fickian behavior [72]. Comparable values have been reported for D4–D6 using inverse gas chromatography and membrane‐based permeation measurements, confirming their high mobility within PDMS matrices [77, 78]. These values are comparatively high for transport through a dense biomedical polymer and consistent with the rubbery state, flexible Si–O–Si backbone, low glass‐transition temperature, high segmental mobility, and transient free volume of PDMS. By comparison, glassy polymers with restricted segmental motion generally exhibit diffusivities for penetrants of comparable size because accessible free volume and chain rearrangement are more limited. The position of PDMS at the opposite end of this spectrum explains why it is mechanically attractive yet intrinsically challenging as a long‐term containment material for chemically similar siloxanes. The experimental data are consistent with free‐volume‐based transport theories, particularly the Vrentas–Duda model [79, 80] that relates diffusivity to polymer segmental dynamics, temperature, penetrant molecular size, and the availability of transient free volume within the matrix. Such models may provide a basis for estimating how crosslink density, membrane composition, filler loading, and temperature influence siloxane transport. A characteristic diffusion timescale may be approximated as *t* ∼ *L* ^2^/*D*, where *L* is the membrane thickness. For a membrane thickness of approximately 0.3–1.0 mm [81] and diffusion coefficients in the range of 10^−^^1^ ^1^ to 10^−^^1^ ^2^ m^2^ s^−^^1^ the corresponding characteristic timescale spans approximately hours to days under idealized one‐dimensional conditions. These estimates describe diffusion through the membrane alone and do not account for sorption, desorption, multilayer resistance, concentration‐dependent transport, or biological interfacial effects. Under steady‐state conditions, the permeation flux is inversely proportional to membrane thickness and proportional to the permeability coefficient and concentration difference across the membrane. Thus, increased membrane thickness or the incorporation of low‐solubility barrier layers can reduce flux without eliminating transport. Diffusion coefficients in this range are compatible with transport over clinically relevant implantation periods, even in the absence of macroscopic defects [82]. However, diffusion coefficients alone do not determine tissue exposure; cumulative transport also depends on penetrant concentration, partition coefficients, membrane thickness, multilayer architecture, and interfacial conditions. For implants intended to remain in the body for decades, even relatively low fluxes may result in measurable cumulative exposure.

### 4.2 Design Strategies to Modulate Permeability in Silicone Elastomers

#### 4.2.1 Gel Cohesiveness and the Mobile Fraction: Crosslink Density Versus Permeability

Silicone gels consist of a crosslinked PDMS network (gel fraction) and a non‐crosslinked mobile fraction containing extractable LMWS (Figure 2C). This mobile fraction is not covalently incorporated into the network and can act as a reservoir of penetrants capable of diffusing under a chemical‐potential or concentration gradient [83, 84]. In this context, the mobile fraction constitutes the primary reservoir driving permeation, as only these species are sufficiently mobile to partition into the surrounding elastomer membrane. While increased crosslink density enhances gel cohesiveness and limits macroscopic gel flow, it does not eliminate the presence of extractable low‐molecular‐weight species, which remain available for diffusion. Clinical and experimental studies report that (i) increased gel cohesiveness does not consistently reduce measurable permeating markers of silicone transfer, (ii) dose‐dependent relationship has been observed between “gel bleed” and capsular stiffness in animal models [85], and (iii) no significant reduction has been reported in tissue silicone detection when comparing cohesive versus non‐cohesive implants [86]. All together, these findings indicate that gel formulation modulates the quantity and release kinetics of mobile species but does not, by itself, determine the overall permeation behavior of the implant.

#### 4.2.2 Multilayered Membrane Architecture and (Retardation) Barrier Layers

To increase resistance to mass transfer, manufacturers have introduced multilayer membrane designs incorporating thicker membranes and low‐permeability barrier layers [87, 88]. From a mass‐transfer perspective, silicone migration can be modeled as resistances in series of transport resistances comprising (1) mobile fraction release from the gel, (2) diffusion across each membrane layer, and (3) interfacial and external resistances associated with the periprosthetic environment. Barrier layers, often composed of chemically modified or fluorinated silicones, may reduce the permeability coefficient, *P* = *D* × *S*, by decreasing penetrant solubility, diffusivity, or both, depending on their composition and microstructure. Thus, multilayer architectures increase transport resistance but do not necessarily eliminate permeation. In that vein, analyses of PIP explants, which exhibited altered membrane composition characterized by the absence of effective barrier layers were associated with markedly increased diffusion potential [89].

#### 4.2.3 Fillers and Tortuosity

Medical‐grade PDMS membranes are typically reinforced with amorphous fumed silica fillers to improve mechanical strength [90]. Silica fillers influence LMWS transport through several mechanisms such as increased tortuosity, modification of polymer free volume, and the formation of polymer‐filler interfacial regions that may either restrict or facilitate molecular motion. These effects highlight that permeability in PDMS is not solely governed by bulk chemistry, but by microstructural organization at the nanoscale. Indeed, studies on PDMS nanocomposites demonstrate that filler incorporation can alter gas and small‐molecule permeability [91]. Depending on filler content, dispersion, and interfacial properties, permeability may decrease (due to tortuosity) or increase (due to interfacial free‐volume effects) (Figure 2C) [92]. However, standardized long‐term diffusion data for medical implant‐grade membranes, remain limited [90]. Consequently, the effects of filler type, concentration, dispersion, and ageing on the long‐term permeability of medical‐grade implant membranes remain insufficiently quantified under physiological conditions.

### 4.3 Time‐Dependent Evolution of Silicone Transport Properties In Vivo

Because implants are intended for long‐term implantation, understanding how their transport properties evolve under physiological conditions is important for assessing long‐term performance.

#### 4.3.1 Aging as a Determinant of Chemical Stability and Surface Evolution

Long‐term implantation exposes PDMS membranes to a biologically active environment that may alter surface chemistry, polymer–filler interactions, solubility, and diffusivity. Nonetheless, explant analyses generally indicate that the bulk chemical and mechanical integrity of PDMS may remain substantially preserved after extended implantation [87, 93]. Some studies have nevertheless reported volatile methylsiloxane release from the membrane itself [94]. In other PDMS systems, accelerated surface aging processes (e.g., plasma treatment, UV‐ozone exposure, environmental conditioning) can modify diffusion properties via formation of silica‐like surface layers or altered free‐volume distributions [95, 96]. Similarly, thermal or environmental aging may induce subtle polymer‐filler interfacial changes without compromising network integrity [97, 98]. To date, no study has directly quantified diffusion coefficients in explanted implants as a function of implantation time, leaving the long‐term evolution of permeation kinetics largely unresolved. This remains an important knowledge gap because relatively subtle changes in free volume or interfacial chemistry may alter long‐term transport behavior even in the absence of gross structural failure.

#### 4.3.2 Desorption and Tissue Interaction: Hydrophobic Gradients and Tissue Accumulation

After diffusing across the intact PDMS membrane, LMWS transfer from the polymer surface into surrounding tissue. This final desorption permeation step is controlled by interfacial partitioning between the implant membrane and the adjacent biological environment. Based on well‐established lipid‐PDMS interactions [99, 100], and consistent with the “like dissolves like” principle, we hypothesize that lipid‐rich breast tissue environment (Figure 2B,D) may establish a favorable partitioning environment for LMWS transfer into the tissue. In this conceptual interpretation, lipid‐rich tissues may promote an outward chemical–diffusion gradient potentializing siloxane permeation. Accordingly, models of silicone transport should consider not only membrane properties but also partitioning at the biological interface. However, direct quantitative in vivo evidence for this mechanism remains limited, and further studies are needed to validate this interpretation.

Explant analyses frequently report yellow or brown discoloration of intact membranes after long‐term implantation (Figure 2D) [101], a phenomenon attributed to the ingress of endogenous lipids into the elastomer matrix. These observations demonstrate bidirectional exchange at the polymer–tissue interface and support the role of lipid‐silicone partitioning gradients during silicone permeation [94, 102]. In addition, immediate adsorption of plasma proteins and lipids following surgery—the initial steps of the FBR‐ and their subsequent maturation over time alter the effective surface chemistry of the PDMS membrane, potentially modulating the progression of the FBR [19, 103]. Notably, adsorbed lipid–protein layers may reduce interfacial resistance and facilitate partitioning of siloxanes into the adjacent tissue although this effect has not yet been directly quantified in vivo (see Section 5.2 “The hidden protein‐lipid interface”). Taken together, the available observations suggest that tissue composition and biomolecular adsorption may influence LMWS desorption and local accumulation, although the magnitude and direction of these effects remain insufficiently quantified under in vivo conditions.

#### 4.3.3 Mechanical Fatigue Versus Permeation: An Unresolved Question

Fatigue phenomena in PDMS include subcritical crack growth, surface microdamage accumulation and gradual changes in mechanical modulus [104]. Explant studies report fatigue‐related structural changes and altered mechanical properties [54, 105]. Simultaneously, the silicone membrane acts as a dynamic interface, functioning as a transient reservoir for bidirectional mass transport: the outward migration of LMWS from the internal gel and the inward ingress of lipids from the periprosthetic environment. Time‐dependent effects of gel‐induced implant membrane swelling have been extensively characterized across first, second, and third‐generation prostheses to elucidate their role in long‐term fatigue and rupture. This influx induces “silicone swelling,” where uptake of mobile silicone species increases the spacing between PDMS polymer chains, alters macromolecular volume and may reduce structural integrity [52, 53]. Furthermore, such swelling may increase free volume and thereby modify membrane permeability, although the magnitude of this effect in clinically used implants remains uncertain. An increase in free volume could also facilitate the ingress of oxygen or reactive species from the inflammatory periprosthetic environment. These observations suggest that silicone permeation, swelling, oxidation, and mechanical fatigue may be coupled during long‐term implantation. However, there is currently no direct evidence linking mechanical fatigue in intact membranes to altered siloxane permeation. Establishing such a relationship would require combined mechanical, structural, and transport measurements under clinically relevant ageing conditions.

## 5 When Silicones Meet Biology

### 5.1 Evidence of In Vivo Transport and Accumulation

Once molecular transport across the polymer membrane is established, the controlling factor is no longer the material alone but the interfacial environment. In vivo characterization of silicone transfer from breast implants to host tissue is primarily characterized in the periprosthetic tissue, the first recipient of silicone implant‐derived products (Figure 3A). By functioning as an integrative reservoir [20], this tissue reflects the cumulative flux of permeating siloxane species over time.

**FIGURE 3:**
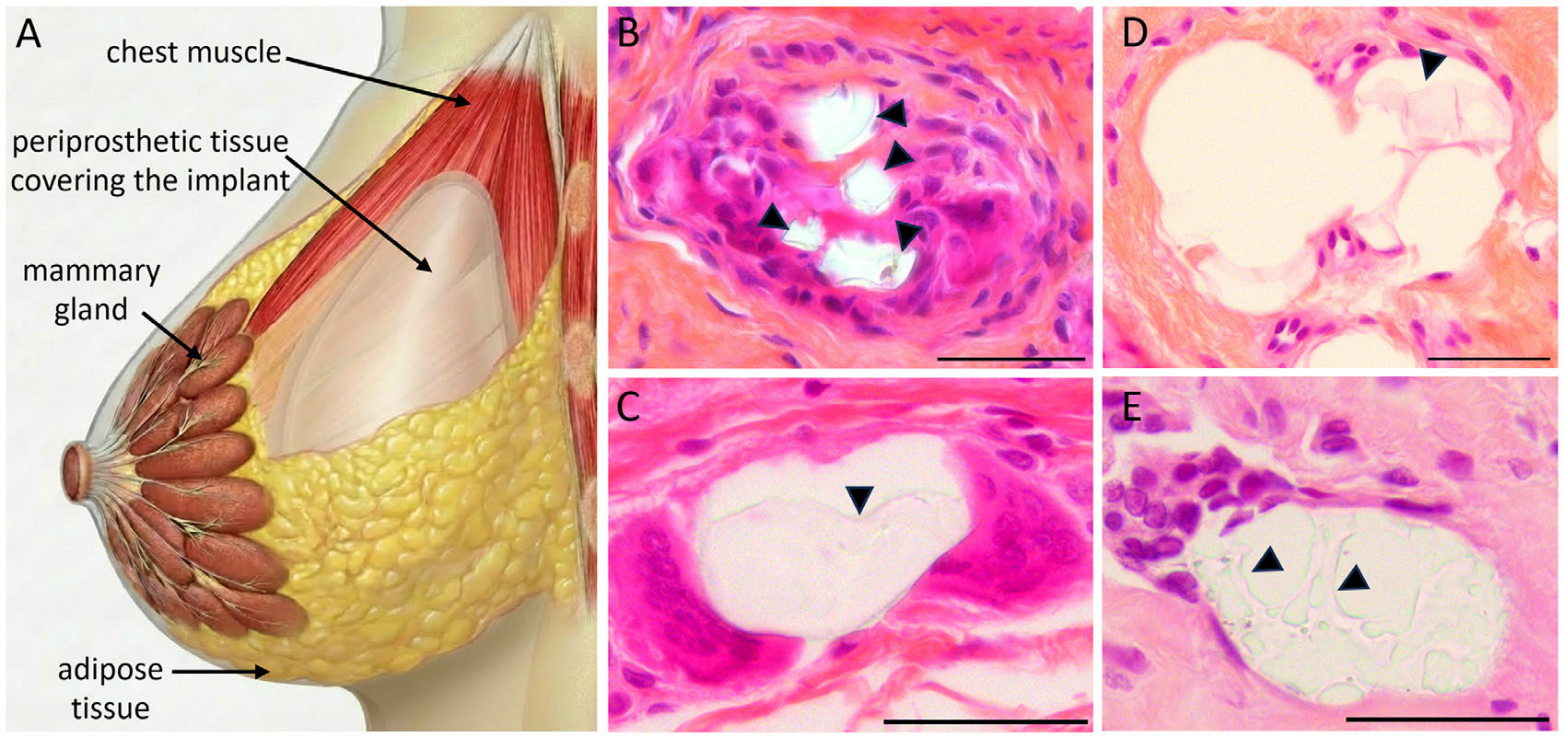
(A) Schematic of the periprosthetic tissue surrounding a silicone breast implant. (B–E) H&E‐stained periprosthetic tissue histological sections showing silicone (black head arrow) surrounded by immune cells (pink nuclei) originating from (B) PDMS implant membrane (serum‐filled ruptured implant), (C and D) surface debris and/or gel permeation (non‐ruptured gel‐filled micro‐ (C) and macrotextured implant (D)), (E) gel filler (gel‐filled ruptured implant). Scale bar: 50 µm (images from personal archives).

Conventional detection is based on hematoxylin and eosin (H&E)‐stained histological sections examined by light microscopy. In such sections, silicone typically appears as optically empty vacuolar inclusions, partially filled with a thread‐like structure or droplets of amorphous, refractile, non‐polarizing materials surrounded by immune cells [62]. Silicone has also been described intracellularly, in the cytoplasm of macrophages or fused macrophages known as foreign body giant cells (Figure 3B–E) [20]. These features are consistent with the phase separation of hydrophobic materials within an aqueous biological environment. However, this approach remains inherently indirect and chemically non‐specific, as it relies on morphological inference rather than molecular identification. Critically, H&E staining does not permit discrimination between distinct polymer state, namely (i) solid crosslinked PDMS debris originating from membrane degradation (Figure 3B), and (ii) liquid LMWS arising from diffusion‐driven permeation (Figure 3C–E). In intact implants, this limitation prevents differentiation between failure‐related debris and intrinsic permeation products, obscuring the underlying transport mechanism (Figure 3C,D). To overcome these limitations, a range of complementary analytical techniques has been developed. Histochemical staining using modified oil Red O (MORO) enhances visualization of hydrophobic species but remains semi‐specific and prone to artefacts due to co‐staining of endogenous lipids [12]. Therefore, MORO staining is more reliable when combined with other analytical methods such as *energy‐dispersive x‐ray spectroscopy (EDX)*.

First applied in 1979 [60] in conjunction with microscopy (polarization, electron or transmission electron microscopy (TEM), EDX enables elemental identification of silicon within MORO‐stained vacuoles, validating the stain's specificity for silicone polymers [12]. However, EDX is technically demanding and limited to specialized facilities. Elemental quantification methods, including inductively coupled plasma techniques (ICP‐HR‐IDMS, ICP‐AES) and graphite furnace atomic absorption spectroscopy (GFAAS), provide high sensitivity for silicon detection and have demonstrated significantly elevated silicon levels in periprosthetic and surrounding tissues of patients with silicone gel implants. ICP‐HR‐IDMS coupled with gas chromatography coupled mass spectrometry (GC‐MS) revealed the presence of D4, D5, and D6 in periprosthetic, muscle and fat tissues from implanted patients, while no siloxanes were detected in control breast tissue samples [75]. Serum from women with silicone gel breast implants analyzed by ICP‐AES for elemental silicon, revealed significantly elevated silicon levels in implanted patients (*n* = 72) compared with control subjects (*n* = 55) [106]. Using the same technics, silicon content was quantified in breast parenchymal and periprosthetic capsular tissues from women with silicone gel or saline implants. These analyses demonstrated markedly higher silicon concentrations in tissues from silicone gel implant patients (mean, 9287 µg/g; *n* = 106) compared with saline‐implant recipients (mean, 196 µg/g; *n* = 37) and nonaugmented control tissues (mean, 64 µg/g; *n* = 17) and that tissue silicon levels correlated with histologic features such as vacuolization and the presence of foamy histiocytes [107]. Complementary GFAAS studies confirmed markedly increased silicon levels in periprosthetic tissues, breast tissue, and regional lymph nodes associated with silicone gel implants, with concentrations varying according to tissue type and proximity to the implant, consistent with local diffusion of silicone‐derived material [108, 109]. These findings support the occurrence of silicone transfer and its spatial distribution in vivo. However, such approaches require tissue digestion and therefore lack spatial resolution, preventing correlation with histological organization.

Spectroscopic methods, including *infrared* (IR) [110] and Raman‐based techniques, enable chemical identification of silicone species with improved specificity have also been used to characterize the chemical identity of silicone inclusions. In particular, *stimulated Raman scattering* (SRS) microscopy offers non‐destructive, high‐resolution imaging compatible with standard histological preparations and capable of submicron resolution imaging [21], often used in combination with H&E [111] or MORO staining [112]. Nevertheless, these techniques remain constrained by limited spectral coverage, instrumentation complexity, or cost. After decades of analytical refinement, there is general acceptance that the refractile vacuolar material observed in H&E‐stained sections corresponds to silicone, supporting quantitative analyses of silicone in periprosthetic tissues [61, 113]. Quantitative image analysis of histological sections has estimated 21–684 silicone droplets/mm^3^ of capsular tissue, with few or no droplet detected around saline‐filled implants, suggesting the gel filler as the primary source [114]. On average, 62 particles per mm^3^ were identified, equivalent to roughly one million particles per periprosthetic tissue. Nonetheless, these values likely underestimate true levels due to silicone loss during routine histological processing, particularly during paraffin embedding and xylene exposure [12, 107, 115]. In that, a recent comparative studies using snap‐frozen samples demonstrated up to fivefold higher silicone retention than conventional methods [115].

Despite these advances, no current method simultaneously satisfies all criteria of specificity, sensitivity, speed, and non‐destructiveness (Figure 4). As a result, a fundamental limitation persists: current analytical approaches detect silicon or bulk hydrophobic material but fail to resolve the molecular identity and physical state of silicone species. From a polymer science perspective, this limitation is critical as permeation is inherently a molecular‐scale transport process, governed by species‐specific properties such as size, volatility, and polymer affinity. However, most analytical methods collapse this complexity into bulk or elemental signals, effectively masking the distinction between diffusion‐driven permeation and particulate release. This creates a mismatch between analytical resolution and transport physics, hindering the ability to establish direct relationships between material structure, molecular transport, and biological response. Accordingly, advancing the understanding of silicone permeation requires the development of integrated analytical strategies capable of resolving both the chemical identity and spatial distribution of silicone species in situ. Bridging this gap is essential for linking polymer transport mechanisms to biological outcomes and for informing the rational design of next‐generation implantable materials.

**FIGURE 4:**
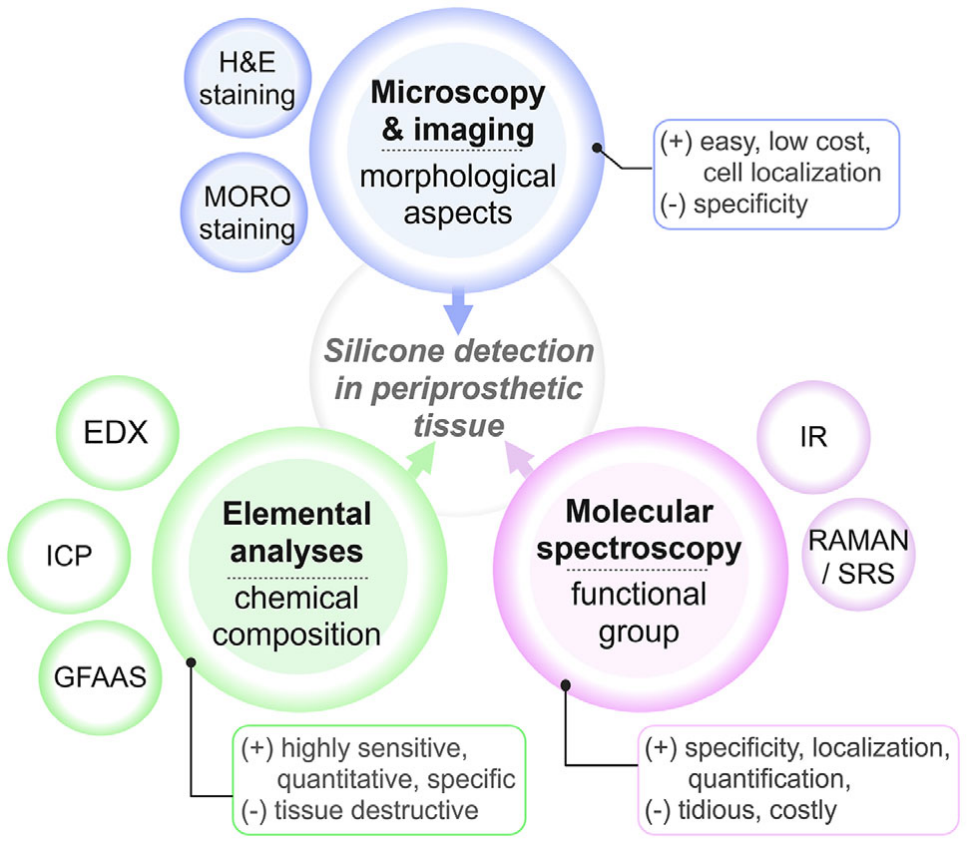
Overview of techniques for identifying and analyzing silicone in periprosthetic tissue, with key advantages and limitations.

### 5.2 The Hidden Protein–Lipid Interface of Silicone Implants

The interface does not just respond to materials: it actively redefines their transport properties. Upon implantation, the layer of adsorbed plasma molecules, including proteins and lipids, forms the original biological interface of the implant that could affect desorption, redistribution, local bioavailability, and interactions with surrounding tissues during biointegration. As a consequence, the biointerface represents a critical regulator shaping both (i) local reactions at the implant site during the foreign body response (FBR) [116], and, potentially, (ii) systemic responses associated with the migration of permeating silicone‐derived species.

#### 5.2.1 Protein–Lipid Interface Layer Shapes Local Silicone–Host Interactions

Immediately after implantation, plasma proteins rapidly adsorb onto the implant surface, establishing the first biologically recognized interface sensed by host cells and initiating the earliest cellular events of the FBR (Figure 2D). Because immune cells do not directly recognize silicone through specific molecular receptors, the composition and organization of the adsorbed proteins further dictate the nature and magnitude of the inflammatory cascade along implantation. For instance, covalent grafting of human serum albumin onto silicone implants significantly mitigates the foreign body response in mice [117]. Additionally, studies using PDMS model surfaces demonstrated that the adsorption kinetics of plasma proteins such as fibrinogen, collagen I, and albumin play a critical role in regulating endothelial cell adhesion [116]. Proteomic analyses of explanted human implants further identified more than 800 surface‐associated proteins, with their relative abundance varying across commercial implant type [103]. Extracellular matrix proteins including fibronectin, fibrinogen, vitronectin, and albumin are among the most prevalent of this adsorbed layer, followed by components of the immune complement system [103]. The biological activity of this protein corona is further influenced by silicone‐specific physicochemical properties. Due to its low surface energy and hydrophobicity, PDMS can promote protein adsorption‐induced conformational rearrangements, potentially exposing previously buried hydrophobic domains and altering protein functionality without necessarily disrupting secondary structure. These structural modifications can affect protein‐cell interactions and downstream signaling pathways involved in FBR progression. Indeed, silicone–protein interactions have been shown to induce conformational changes and even aggregation, as previously demonstrated for myoglobin adsorbed onto PDMS [118]. Importantly, these effects depend on both protein identity and siloxane molecular structure. For example, the tertiary structure of fibrinogen is altered by cyclic D3, short‐chain L2, and long‐chain PDMS [119, 120]. D3 and short‐chain PDMS interact with collagen, reducing fibrillogenesis without denaturing native structure; instead, collagen is sequestered within interlayers of collagen‐siloxane complexes [121]. Similarly, LMWS, especially D4, facilitate human serum albumin denaturation and subsequent aggregation [122]. Accordingly, these observations indicate that silicone does not directly trigger biological responses, but rather reshapes the biological identity of the implant by modulating the structure and function of adsorbed endogenous proteins, thereby converting host biomolecules into mediators of the FBR. Mechanistically, protein conformational rearrangements at the PDMS interface may stabilize adsorption onto the low‐surface‐energy polymer while altering ligand accessibility, cellular recognition, and subsequent inflammatory signaling. In contrast to the well‐characterized role of the protein corona in modulating the FBR, the contribution of the lipid interface remains less well understood. Nevertheless, emerging studies have shown that differences in lipid deposition patterns are associated with variations in FBR and immune responses [19, 123], highlighting the potential importance of the lipid interface as an area for future investigation.

#### 5.2.2 Lipoprotein‐Mediated Transport as a Hypothesis for Systemic LMWS Redistribution

Beyond their role in shaping inflammation, proteins may also facilitate systemic transport of silicone permeates. While silicone permeates are primarily confined to the periprosthetic tissue, they have been detected in more distant tissues including intramammary lymph nodes and other lipophilic organs (e.g., liver, spleen, or brain [12, 22]) where they have been associated with silicone‐related inflammatory responses, sometimes persisting even after implant removal [124, 125]. Given their high hydrophobicity, silicone‐derived species are expected to preferentially associates with lipid structures rather than remain freely dissolved in aqueous fluids. In this context, lipid‐associated carriers such as lipoproteins represent plausible mediators of silicone redistribution, as they are physiologically involved in the transport of hydrophobic molecules within aqueous environments such as blood and lymphatic fluids [126]. Supported by the detection of silicone in regional lymph nodes [21, 127], lipid‐associated vectors would carry silicone from the breast pocket into lymphatic drainage pathways and the bloodstream, where they may disseminate systemically and modulate distant immune responses. This suggests that silicone dissemination is governed by coupled transport mechanisms combining polymer partitioning, protein binding and lipid‐mediated trafficking rather than simple passive diffusion. Several lines of indirect evidence support this hypothesis. Analogous carrier‐mediated mechanisms have been well documented for hydrophobic xenobiotics, including volatile silicones used in cosmetic formulations [128] and persistent environmental contaminants such as PFAS [129], which can associate with plasma lipoproteins and promote foam cell formation. Furthermore, excessive uptake of lipid‐associated complexes can promote intracellular lipid accumulation and foam‐like phenotypes [130, 131, 132], a hallmark feature observed in silicone‐exposed periprosthetic tissues [11, 20, 62]. In addition, interactions between lipids and silicone materials may be bidirectional, as lipid infiltration (including oxylipin accumulation) has been implicated in silicone breast implant elastomer degradation and implant aging [19, 94, 102, 133] suggesting early interactions between lipids entering the silicone matrix and LMWS retained within the gel phase. Finally, lipid deposition has been documented in other silicone‐based devices such as contact lenses [134, 135], suggesting that lipid‐mediated silicone transport represents a broader phenomenon applicable across a range of silicone‐based implants. Therefore, these observations support a lipid‐assisted transport paradigm in which lipoprotein‐associated mechanisms may contribute to silicone redistribution from the implant pocket to distant tissues. However, although mechanistically plausible, pathways for systemic silicone dissemination remain largely underexplored. Limited access to human tissues, the absence of standardized experimental models, and methodological differences in silicone detection have hindered definitive conclusions. While some studies have reported silicone accumulation in regional and distant tissues from intact implants, other investigations have found little evidence of clinically significant systemic dissemination, and the extent and biological relevance of silicone migration remain controversial [136]. These discrepancies may reflect differences in implant characteristics, analytical approaches, and patient populations. Further longitudinal studies with standardized analytical techniques are needed to clarify the mechanisms and biological relevance of systemic silicone exposure and resolve this long‐lasting controversy.

## 6 Permeation as a Shared Property of Polymeric Materials

The permeation behavior observed in silicone breast implants can be situated within the established framework governing molecular transport in soft materials in vivo (Figure 5). Silicone permeation therefore represents a clinically relevant example of how polymer structure, penetrant affinity, and thermodynamic driving forces govern molecular transport through soft materials. Across a wide spectrum of biomedical polymers, including elastomers, hydrogels, drug delivery matrices, and biological gels, permeability is governed by established principles of polymer transport. In all cases, transport is governed by the coupled contributions of solubility and diffusivity, which are themselves determined by the coupling between polymer structure, free volume, chain mobility, chemical affinity between the penetrant and the polymer phase and the surrounding biological environment. These transport principles are shared across a wide class of hydrated and non‐hydrated polymer systems, including hydrogels, drug delivery matrices, and other implantable devices. In hydrogels, for instance, permeability is governed by the interplay between network mesh size, polymer–solute interactions, and environmental stimuli, leading to highly tunable relationships between partitioning and diffusivity, commonly expressed as *P* = *K* × *DP* = *K* ∖times *DP* = *K* × *D*) where *K* represents the partition coefficient [137]. Importantly, these systems demonstrate that permeability is inherently dynamic: swelling, degradation, and environmental stimuli continuously reshape transport pathways over time (Figure 5). Comparable time‐dependent effects may occur in silicone elastomers, where lipid uptake, interfacial biomolecular adsorption, and aging modify interfacial properties, effective free volume, and diffusivity [137].

**FIGURE 5:**
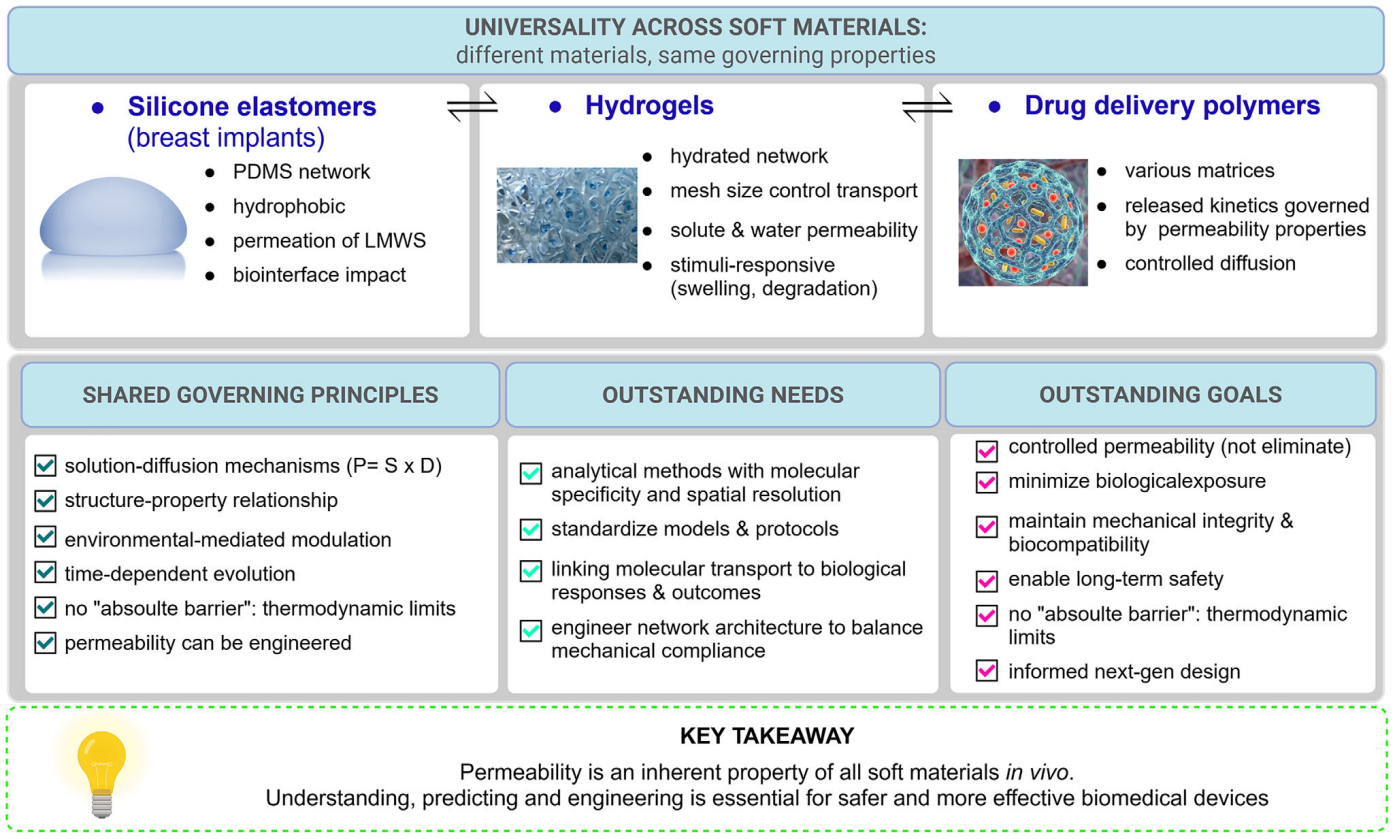
Permeation as a shared property of polymeric materials.

Similarly, in drug delivery polymers, permeability is not a defect but is often deliberately engineered to achieve controlled release. In such systems, diffusion through polymer matrices, and may be described using related diffusion and partitioning principles, with release kinetics governed by polymer relaxation, penetrant solubility, and matrix degradation. The key distinction is conceptual: whereas permeability in implants is often interpreted as a failure mode, in drug delivery it is recognized as an intrinsic and tunable material function. This comparison highlights how molecular transport should be viewed either as an undesirable release pathway or as a deliberately engineered function, depending on the intended application. Silicone‐based systems also provide direct examples in which permeability is exploited as a functional property. In silicone‐hydrogel contact lenses, oxygen transport through the silicone‐rich phase is deliberately optimized to support corneal oxygenation [138, 139, 140], while silicone‐based membranes and matrices have been widely investigated for controlled transdermal and implantable drug delivery [141, 142, 143]. These examples illustrate that the high permeability of silicone materials can be advantageous when molecular transport is appropriately characterized and controlled, contrasting with applications in which molecular containment is the primary objective. In biological gels such as mucus or extracellular matrices, selective permeability emerges from dynamic interactions between penetrants and the network, including transient binding to crosslinks, further emphasizing that transport may involve both diffusion and reversible interactions with the surrounding network [144]. Across these systems, permeability is a multiscale and environment‐dependent property shaped by structural heterogeneity, physicochemical affinity, and time‐dependent remodeling of the material (Figure 5). Within this broader context, silicone permeation represents one specific application of established soft‐material transport principles under physiological conditions. Viewing silicone implants within this broader transport framework shifts the focus from attempting to eliminate permeability toward understanding, predicting, and ultimately engineering it across classes of biomedical materials. This includes designing materials with tailored transport properties, developing interface‐aware strategies to mitigate biological interactions, and improving analytical tools capable of resolving molecular transport processes in situ. Situating silicone implants within the established and broader landscape of soft material permeability clarifies physicochemical basis of gel bleed and highlights parameters that may be targeted in future implant design, including polymer architecture, mobile‐species content, barrier‐layer composition, and biological interfacial interactions [145]. At the same time, the comparisons with hydrogels, drug‐delivery matrices, and other silicone‐based devices illustrate that permeability is application‐dependent: it may be intentionally exploited in controlled‐release or oxygen‐transport systems, but requires careful limitation and characterization where molecular containment is desired.

## 7 Conclusion

Silicone permeation from breast implants remains a largely unresolved challenge. Despite early recognition as a key limitation to long‐term implantation, the underlying mechanisms, from molecular speciation and release at the implant surface to transport within peri‐implant and distant tissues, are still insufficiently characterized. This gap reflects the intrinsic complexity of silicone permeation, which spans polymer physics, interfacial chemistry, and biological response, as well as the challenges of capturing both the chemical diversity and in vivo transport dynamics of silicone species. Interfacial phenomena further complicate this picture. The rapid formation of a dynamic biomolecular layer of proteins and lipids at the implant surface redefines the effective interface, modulating diffusion gradients, partitioning behavior, and desorption kinetics in the biological milieu. In this context, silicone permeation should be viewed not as an isolated process but as an emergent property of implant design, linking molecular architecture to transport behavior, tissue exposure, and ultimately biological response. This systems‐level perspective provides a foundation for a shared mechanistic framework. Importantly, this framework shifts the design paradigm from attempting to eliminate permeability toward understanding, predicting, and ultimately controlling it across classes of biomedical materials. Looking forward, emerging analytical and computational tools offer unprecedented opportunities to close this gap. Advances in high‐resolution spectroscopy, multimodal imaging and in situ chemical mapping are beginning to enable more precise identification and quantification of silicone species within complex biological environments. Future research should prioritize the direct in vivo quantification of silicone permeation kinetics over clinically relevant implantation periods, comprehensive characterization of the molecular speciation of silicone compounds in peri‐implant and distant tissues, and the development of standardized analytical methodologies capable of distinguishing molecular permeation products from particulate silicone debris. At the same time, artificial intelligence and data‐driven approaches are poised to transform the field [3]. Machine learning models offer new opportunities to integrate heterogeneous datasets, including polymer composition, transport parameters, imaging outputs, and clinical observations, to better understand and predict permeation behavior. AI‐assisted image analysis may enhance the detection and quantification of silicone in histological samples, overcoming limitations of conventional staining methods. Furthermore, the integration of multiscale modeling combining molecular simulations with continuum transport and data‐driven approaches offers a path toward predictive design of next‐generation implant materials. By linking polymer structure, transport behavior, and biological response, these strategies may enable the rational development of implants that balance mechanical performance, biocompatibility, and controlled molecular permeability. Therefore, advancing the understanding of silicone permeation will require continued technological innovation together with interdisciplinary research integrating polymer science, analytical chemistry, materials science, and biology. Combining improved experimental methodologies with predictive computational approaches will be essential to establish quantitative relationships between implant design, molecular transport, and biological response. More broadly, this work highlights that permeability should be considered an inherent and tunable material property arising from polymer structure and environmental coupling rather than being view solely as an indicator of material failure. Recognizing this principle may contribute to the rational design of next‐generation implantable materials.

## Author Contributions

I. Brigaud: conceptualization, supervision, writing – original draft, literature search, data curation, figure creation, funding acquisition. H. Mutlu and D. Bouyer: conceptualization, critical review of literature, writing – review and editing the original draft. All authors: conceptual development, synthesis of evidence from the literature and critical revision of the manuscript. All authors approved the final version and agree to be accountable for all aspects of the work.

## Funding

This work was supported by the Agence Nationale de la Recherche (ANR) under grant “Safe‐implant” (ANR‐22‐CPJ1‐0077‐01).

## Ethics Statement

The authors declare no competing financial or non‐financial interests. Funding sources supporting the authors’ research activities are acknowledged separately where applicable. Permissions for reproduction of previously published figures or materials have been obtained from the respective copyright holders where required. This article does not report on any clinical trial, and therefore clinical trial registration is not applicable.

## Conflicts of Interest

The authors declare no conflicts of interest.

## Data Availability

Data sharing not applicable to this article as no datasets were generated or analyzed during the current study.

## References

[1] D. Perry and J. Frame , “The History and Development of Breast Implants,” Annals of The Royal College of Surgeons of England 102 (2020): 478–482, 10.1308/rcsann.2020.0003. PMC7450417 31964154

[2] ISAPS International Survey on (accessed February 12, 2025), www.isaps.org.

[3] K. Dzobo , N. Khumalo , V. Z. Mora , A. Zoncsich , R. de Mezerville , and A. Bayat , “Advances in Silicone Implants Characterization: A Comprehensive Overview of Chemical,” Physical and Biological Methods for Biocompatibility Assessment, Bioengineering 12 (2025): 1307, 10.3390/bioengineering12121307. PMC12729539 41463604

[4] D. E. Katsoulis , R. G. Schmidt , and G. A. Zank , “Siloxanes and Silicones (Advances in Silicone Technologies 2000–15),” in Organosilicon Compounds (Elsevier, 2017), 301–322, 10.1016/B978-0-12-814213-4.00007-1.

[5] M. A. Brook , “The Chemistry and Physical Properties of Biomedical silicones,” in Biomaterials in Plastic Surgery (Elsevier, 2012), 52–67, 10.1533/9780857096418.52.

[6] T. Shimura , Y. Jin , K. Kubyama , et al., “Improved Thermal Properties of Polydimethylsiloxane by Copolymerization and Thiol–ene Crosslinking of 2‐Pyrone‐4,6‐Dicarboxylic Acid Moiety,” Polymer Chemistry 16 (2025): 45–51, 10.1039/D4PY01051E.

[7] G. Deshpande and M. E. Rezac , “Kinetic Aspects of the Thermal Degradation of Poly(dimethyl siloxane) and Poly(dimethyl diphenyl siloxane),” Polymer Degradation and Stability 76 (2002): 17–24, 10.1016/S0141-3910(01)00261-0.

[8] D. E. Barker , M. I. Retsky , and S. Schultz , ““Bleeding” of Silicone From Bag‐Gel Breast Implants, and Its Clinical Relation to Fibrous Capsule Reaction,” Plastic and Reconstructive Surgery 61 (1978): 836–841, http://www.ncbi.nlm.nih.gov/pubmed/351637. 351637

[9] D. E. Barker and S. L. Schultz , “Reaction to Silicone Implants in the Guinea Pig,” Aesthetic Plastic Surgery 1 (1976): 371–379, 10.1007/BF01570272. 24173763

[10] C. Al Sammour , M. Dziubek , M. A. Danino , and S. Ortiz , “Silicone Particles in Breast Implant Capsules: A Retrospective Single‐Center Study Evaluating Silicone Bleeding,” Annales de Chirurgie Plastique Esthétique 70 (2025): 324–334, 10.1016/j.anplas.2025.02.006. 40082135

[11] H. Busch , “Silicone Toxicology,” Seminars in Arthritis and Rheumatism 24 (1994): 11–17, 10.1016/0049-0172(94)90104-X. 7801134

[12] R. M. Kappel , “Gel Bleed and Rupture of Silicone Breast Implants Investigated by Light‐, Electron Microscopy and Energy Dispersive X‐Ray Analysis of Internal Organs and Nervous Tissue,” Clinical Medical Reviews and Case Reports 3 (2016): 10–12, 10.23937/2378-3656/1410087.

[13] N. J. Hallab , L. Samelko , and D. Hammond , “Particulate Debris Released From Breast Implant Surfaces is Highly Dependent on Implant Type,” Aesthetic Surgery Journal 41 (2021): NP782–NP793, 10.1093/asj/sjab051. 33564817

[14] L. H. Webb , V. L. Aime , A. Do , K. Mossman , and R. C. Mahabir , “Textured Breast Implants: A Closer Look at the Surface Debris Under the Microscope,” Plastic Surgery 25 (2017): 179–183, 10.1177/2292550317716127. PMC5626211 29026824

[15] C. Garabedian , R. Vayron , N. Bricout , R. Deltombe , K. Anselme , and M. Bigerelle , “In Vivo Damage Study of Different Textured Breast Implants,” Biotribology 23 (2020): 100133, 10.1016/j.biotri.2020.100133.

[16] E. Swanson , “BIA‐ALCL: Comparing the Risk Profiles of Smooth and Textured Breast Implants,” Aesthetic Plastic Surgery 47 (2023): 245–250, 10.1007/S00266-023-03329-8/METRICS. PMC10439047 36997735

[17] A. K. Groth and R. Graf , “Breast Implant‐Associated Anaplastic Large Cell Lymphoma (BIA‐ALCL) and the Textured Breast Implant Crisis,” Aesthetic Plastic Surgery 44 (2020): 1–12, 10.1007/s00266-019-01521-3. 31624894

[18] J. M. Anderson , A. Rodriguez , and D. T. Chang , “Foreign Body Reaction to Biomaterials,” Seminars in Immunology 20 (2008): 86–100, 10.1016/J.SMIM.2007.11.004. PMC2327202 18162407

[19] C. C. Schreib , M. I. Jarvis , T. Terlier , et al., “Lipid Deposition Profiles Influence Foreign Body Responses,” Advanced Materials 35 (2023): 2205709, 10.1002/ADMA.202205709. PMC10309593 36871193

[20] I. Pluvy , E. Randrianaridera , I. Tahmaz , et al., “Breast Implant Silicone Exposure Induces Immunogenic Response and Autoimmune Markers in Human Periprosthetic Tissue,” Biomaterials 317 (2025): 123025, 10.1016/j.biomaterials.2024.123025. 39719745

[21] Z. C. Feng , K. T. Yue , W. Lu , J. Yiin , B. Klein , and I. T. Ferguson , “Raman Spectroscopic Detection of Silicone Leakage in Human Breast and Lymph Node Tissues,” Journal of Biomedical Research & Environmental Sciences 4 (2023): 860–863, 10.37871/JBRES1745.

[22] M. Win , M. S. Hlaing , W. Htet , et al., “Migration of Silicone Particles into the Liver Following Rupture of Silicone Breast Implant: A Case Report and Literature Review,” Cureus 18 (2026): 101747, 10.7759/CUREUS.101747. PMC12908865 41704994

[23] P. Montemurro , T. Pellegatta , H. Burton , and G. Pafitanis , “Silicone Migration From Breast Implants: A Case of Ocular Siliconoma and Literature Review,” Aesthetic Surgery Journal 43 (2023): 972–977, 10.1093/ASJ/SJAD086. 36991214

[24] S. Azahaf , K. A. Spit , C. J. M. De Blok , P. Bult , and P. W. B. Nanayakkara , “The Role of Positron Emission Tomography Imaging in Breast Implant Illness,” Plastic and Reconstructive Surgery—Global Open 13 (2025): 6458, 10.1097/GOX.0000000000006458. PMC11737493 39823024

[25] Saline, Silicone Gel, and Alternative Breast Implants Guidance for Industry and Food and Drug Administration Staff Preface Public Comment (accessed October 24, 2025), https://www.regulations.gov.

[26] J. W. Cohen Tervaert , N. Mohazab , D. Redmond , C. van Eeden , and M. Osman , “Breast Implant Illness: Scientific Evidence of Its Existence,” Expert Review of Clinical Immunology 18 (2022): 15–29, 10.1080/1744666X.2022.2010546. 34882509

[27] J. W. C. Tervaert , Y. Shoenfeld , C. Cruciani , C. Scarpa , and F. Bassetto , “Breast Implant Illness: Is it Causally Related to Breast Implants?,” Autoimmunity Reviews 23 (2024): 103448, 10.1016/J.AUTREV.2023.103448. 37714420

[28] D. A. Magno‐Padron , J. Luo , T. C. Jessop , et al., “A Population‐Based Study of Breast Implant Illness,” Archives of Plastic Surgery 48 (2021): 353–360, 10.5999/aps.2020.02117. PMC8342259 34352944

[29] E. F. C. Fleury , “The Contradiction of Breast Implant Illness,” Gland Surgery 10 (2021): 2081–2083, 10.21037/gs-21-135. PMC8258878 34268094

[30] J. Kaplan and R. Rohrich , “Breast Implant Illness: A Topic in Review,” Gland Surgery 10 (2021): 430–443, 10.21037/GS-20-231. PMC7882356 33634001

[31] F. Santanelli di Pompeo , G. Paolini , G. Firmani , and M. Sorotos , “History of Breast Implants: Back to the Future,” JPRAS Open 32 (2022): 166–177, 10.1016/j.jpra.2022.02.004. PMC9006741 35434240

[32] G. P. Maxwell and A. Gabriel , “The Evolution of Breast Implants,” Plastic and Reconstructive Surgery 134 (2014): 12S–17S, 10.1097/PRS.0000000000000348. 25057743

[33] B. S. Freeman , “Successful Treatment of Some Fibrous Envelope Contractures Around Breast Implants,” Plastic and Reconstructive Surgery 50 (1972): 107–113, 10.1097/00006534-197208000-00001. 5045376

[34] O. Asplund , “Capsular Contracture in Silicone Gel and Saline‐Filled Breast Implants After Reconstruction,” Plastic and Reconstructive Surgery 73 (1984): 270–275, 10.1097/00006534-198402000-00022. 6695025

[35] S. P. Bartlett , “Polyurethane‐Coated Breast Implant,” Plastic and Reconstructive Surgery 75 (1985): 279, 10.1097/00006534-198502000-00027. 3969417

[36] G. S. Brody , “Fact and Fiction About Breast Implant “Bleed”,” Plastic and Reconstructive Surgery 60 (1977): 615–616, 10.1097/00006534-197710000-00020. 909974

[37] C. Kaoutzanis , J. Winocour , J. Unger , A. Gabriel , and G. P. Maxwell , “The Evolution of Breast Implants,” Seminars in Plastic Surgery 33 (2019): 217–223, 10.1055/s-0039-1696985. PMC6797490 31632203

[38] H. Spiera , “Scleroderma After Silicone Augmentation Mammoplasty,” JAMA: The Journal of the American Medical Association 260 (1988): 236, 10.1001/jama.1988.03410020102037. 3385898

[39] J. Sánchez‐Guerrero , G. A. Colditz , E. W. Karlson , D. J. Hunter , F. E. Speizer , and M. H. Liang , “Silicone Breast Implants and the Risk of Connective‐Tissue Diseases and Symptoms,” New England Journal of Medicine 332 (1995): 1666–1670, 10.1056/nejm199506223322502. 7760867

[40] S. Hoa , K. Milord , M. Hudson , S. C. Nicolaidis , and J. Bourré‐Tessier , “Risk of Rheumatic Disease in Breast Implant Users: A Qualitative Systematic Review,” Gland Surg 10 (2021): 2557, 10.21037/GS-21-266. PMC8411075 34527567

[41] V. Arpino , M. Brock , S. E. Gill , et al., “Capsular Contracture After Breast Augmentation: An Update for Clinical Practice,” Archives of Physiology and Biochemistry 116 (2015): 532–543, 10.1038/onc.2013.568. PMC4579163 26430623

[42] T. R. Hester , N. F. Ford , P. J. Gale , et al., “Measurement of 2,4‐Toluenediamine in Urine and Serum Samples From Women With Même or Replicon Breast Implants,” Plastic and Reconstructive Surgery 100 (1997): 1291–1298, 10.1097/00006534-199710000-00035. 9326795

[43] C. Batich , J. Williams , and R. King , “Toxic Hydrolysis Product From a Biodegradable Foam Implant,” Journal of Biomedical Materials Research 23 (1989): 311–319, 10.1002/JBM.820231406. 2613741

[44] C. Paletta , F. X. Paletta , and F. X. Paletta , “Squamous Cell Carcinoma Following Breast Augmentation,” Annals of Plastic Surgery 29 (1992): 425–432, 10.1097/00000637-199211000-00009. 1444131

[45] N. M. Cole , “Consequences of the U.S. Food and Drug Administration–Directed Moratorium on Silicone Gel Breast Implants: 1992 to 2006,” Plastic & Reconstructive Surgery 141 (2018): 1137–1141, 10.1097/PRS.0000000000004284. 29697610

[46] F. M. Lampert , M. Schwarz , S. Grabin , and G. B. Stark , “The “PIP Scandal”—Complications in Breast Implants of Inferior Quality: State of Knowledge, Official Recommendations and Case Report,” Geburtshilfe Und Frauenheilkunde 72 (2012): 243, 10.1055/S-0031-1298323. PMC4168359 25308983

[47] J. A. Keech and B. J. Creech , “Anaplastic T‐Cell Lymphoma in Proximity to a Saline‐Filled Breast Implant,” Plastic & Reconstructive Surgery 100 (1997): 554–555, 10.1097/00006534-199708000-00065. 9252643

[48] Update on the Safety of Silicone Gel‐Filled Breast Implants (2011)—Executive Summary | FDA, (n.d.) (accessed February 5, 2025), https://www.fda.gov/medical‐devices/breast‐implants/update‐safety‐silicone‐gel‐filled‐breast‐implants‐2011‐executive‐summary.

[49] S. H. Swerdlow , E. Campo , S. A. Pileri , et al., “The 2016 Revision of the World Health Organization Classification of Lymphoid Neoplasms,” Blood 127 (2016): 2375–2390, 10.1182/BLOOD-2016-01-643569. PMC4874220 26980727

[50] S. S. Lajevardi , P. Rastogi , D. Isacson , and A. K. Deva , “What are the Likely Causes of Breast Implant Associated Anaplastic Large Cell Lymphoma (BIA‐ALCL)?,” JPRAS Open 32 (2022): 34–42, 10.1016/J.JPRA.2021.11.006. PMC8867047 35242986

[51] UPDATE: Reports of Squamous Cell Carcinoma (SCC) in the Capsule Around Breast Implants—FDA Safety Communication | FDA, (n.d.) (accessed March 7, 2025), https://www.fda.gov/medical‐devices/safety‐communications/update‐reports‐squamous‐cell‐carcinoma‐scc‐capsule‐around‐breast‐implants‐fda‐safety‐communication.

[52] S. Necchi , D. Molina , S. Turri , F. Rossetto , M. Rietjens , and G. Pennati , “Failure of Silicone Gel Breast Implants: Is the Mechanical Weakening Due to Shell Swelling a Significant Cause of Prostheses Rupture?,” Journal of the Mechanical Behavior of Biomedical Materials 4 (2011): 2002–2008, 10.1016/j.jmbbm.2011.06.019. 22098899

[53] C. J. Wolf , K. L. Jerina , H. J. Brandon , and V. L. Young , “The Effect of Cyclic Swelling (Octamethylcyclotetrasiloxane) on the Physical Properties of Silicone Breast Implant Shells,” Journal of Biomaterials Science, Polymer Edition 13 (2002): 27–41, 10.1163/156856202753525918. 12003073

[54] N. G. Ramião , P. S. Martins , M. L. Barroso , D. C. Santos , and A. A. Fernandes , “An Experimental Analysis of Shell Failure in Breast Implants,” Journal of the Mechanical Behavior of Biomedical Materials 72 (2017): 22–28, 10.1016/j.jmbbm.2017.04.005. 28437660

[55] C. A. Glicksman , “Implant Shell Characteristics,” Clinics in Plastic Surgery 48 (2021): 79–86, 10.1016/J.CPS.2020.09.006. 33220907

[56] P. H. J. Keizers , M. J. Vredenbregt , F. Bakker , D. de Kaste , and B. J. Venhuis , “Chemical Fingerprinting of Silicone‐Based Breast Implants,” Journal of Pharmaceutical and Biomedical Analysis 102 (2015): 340–345, 10.1016/J.JPBA.2014.09.008. 25459933

[57] F. Bassetto , C. Scarpa , E. Caccialanza , M. C. Montesco , and P. Magnani , “Histological Features of Periprosthetic Mammary Capsules: Silicone vs. Polyurethane,” Aesthetic Plastic Surgery 34 (2010): 481–485, 10.1007/s00266-010-9483-0. 20229111

[58] S. V. M. Maharaj , “Platinum Concentration in Silicone Breast Implant Material and Capsular Tissue by ICP‐MS,” Analytical and Bioanalytical Chemistry 380 (2004): 84–89, 10.1007/s00216-004-2714-y. 15243733

[59] L. Pantanowitz and K. Balogh , “Breast Implant Capsule With Synovial Metaplasia,” Breast Journal 9 (2003): 428, 10.1046/j.1524-4741.2003.09518.x. 12968969

[60] M. A. Mandel and D. F. Gibbons , “The Presence of Silicone in Breast Capsules,” Aesthetic Plastic Surgery 3 (1979): 219–225, 10.1007/BF01577858. 24173992

[61] M. A. Danino , M. Dziubek , J. Dalfen , et al., “Silicone Particles in Capsules Around Breast Implants: An Investigation Into Currently Available Implants in North America,” Aesthetic Surgery Journal 44 (2024): 363–372, 10.1093/asj/sjad363. 38078367

[62] P. J. Van Diest , W. H. Beekman , and J. J. Hage , “Pathology of Silicone Leakage From Breast Implants,” Journal of Clinical Pathology 51 (1998): 493–497, 10.1136/jcp.51.7.493. PMC500799 9797723

[63] A. J. Ryu , K. N. Glazebrook , N. Samreen , P. R. Bauer , E. S. Yi , and J. H. Ryu , “Spectrum of Chronic Complications Related to Silicone Leakage and Migration,” American Journal of Medicine 131 (2018): 1383–1386, 10.1016/j.amjmed.2018.03.041. 29680487

[64] A. Larsen , E. E. F. Bak , L. B. Hart , et al., “Silicone Leakage From Breast Implants is Determined by Silicone Cohesiveness: A Histologic Study of 493 Patients,” Plastic & Reconstructive Surgery 154 (2024): 1159–1171, 10.1097/PRS.0000000000011395. 39591364

[65] R. El‐Haddad , B. Lafarge‐Claoue , C. Garabedian , and S. Staub , “A 10‐Year Prospective Study of Implant‐Based Breast Augmentation and Reconstruction,” Eplasty 18 (2018): 7, http://www.ncbi.nlm.nih.gov/pubmed/29487671. PMC5807774 29487671

[66] J. Daenicke , D. W. Schubert , M. Hedenqvist , E. Linde , T. Sigl , and R. E. Horch , “Evaluation of the Influence of Crosslink Density and Penetrant Size on the Diffusion Properties of Silicone Oils Into Silicone Elastomers,” in AIP Conf. Proc. (AIP Publishing LLC AIP Publishing, 2019), 080004, 10.1063/1.5084878.

[67] I. De Bo , H. Van Langenhove , P. Pruuost , et al., “Investigation of the Permeability and Selectivity of Gases and Volatile Organic Compounds for Polydimethylsiloxane Membranes,” Journal of Membrane Science 215 (2003): 303–319, 10.1016/S0376-7388(03)00024-3.

[68] T. C. Merkel , V. Bondar , K. Nagai , B. D. Freeman , and P. Y. Yampolskii , “Gas Sorption, Diffusion, and Permeation in Poly(2,2‐bis(trifluoromethyl)‐4,5‐difluoro‐1,3‐dioxole‐co‐tetrafluoroethylene),” Macromolecules 32 (1999): 8427–8440, 10.1021/MA990685R.

[69] S. Kanehashi and K. Nagai , “Gas and Vapor Transport in Membranes,” Membrane Characterization 3 (2017): 309–336, 10.1016/B978-0-444-63776-5.00014-0.

[70] L. M. Robeson , “The Upper Bound Revisited,” Journal of Membrane Science 320 (2008): 390–400, 10.1016/J.MEMSCI.2008.04.030.

[71] E. Favre , P. Schaetzel , Q. T. Nguygen , R. Clément , and J. Néel , “Sorption, Diffusion and Vapor Permeation of Various Penetrants Through Dense Poly(dimethylsiloxane) Membranes: A Transport Analysis,” Journal of Membrane Science 92 (1994): 169–184, 10.1016/0376-7388(94)00060-3.

[72] C. J. Wolf , K. L. Jerina , H. J. Brandon , and V. L. Young , “The Transport of Octamethylcyclotetrasiloxane (D4) and Polydimethylsiloxane (PDMS) in Lightly Cross‐Linked Silicone Rubber,” Journal of Biomaterials Science, Polymer Edition 12 (2001): 801–815, 10.1163/156856201750411675. 11587042

[73] E. D. Lykissa , S. V. Kala , J. B. Hurley , and R. M. Lebovitz , “Release of Low Molecular Weight Silicones and Platinum From Silicone Breast Implants,” Analytical Chemistry 69 (1997): 4912–4916, 10.1021/ac970710w. 9406537

[74] D. Flassbeck , B. Pfleiderer , R. Grümping , and A. V. Himer , “Determination of Low Molecular Weight Silicones in Plasma and Blood of Women After Exposure to Silicone Breast Implants by GC/MS,” Analytical Chemistry 73 (2001): 606–611, 10.1021/AC000738Z. 11217769

[75] D. Flassbeck , B. Pfleiderer , P. Klemens , K. G. Heumann , E. Eltze , and A. V. Hirner , “Determination of Siloxanes, Silicon, and Platinum in Tissues of Women With Silicone Gel‐Filled Implants,” Analytical and Bioanalytical Chemistry 375 (2003): 356–362, 10.1007/S00216-002-1694-Z. 12589499

[76] P. Rosendahl , J. Hippler , O. J. Schmitz , O. Hoffmann , and P. Rusch , “Cyclic Volatile Methylsiloxanes in Human Blood as Markers for Ruptured Silicone Gel‐Filled Breast Implants,” Analytical and Bioanalytical Chemistry 408 (2016): 3309–3317, 10.1007/S00216-016-9401-7. 26968566

[77] R. E. Baltus , M. M. Alger , and T. J. Stanley , “Solubility and Diffusivity of Cyclic Oligomers in Poly(dimethylsiloxane) Using Capillary Column Inverse Gas Chromatography,” Macromolecules 26 (1993): 5651–5656, https://pubs.acs.org/sharingguidelines.

[78] H. A. Balogun and R. P. Lively , “Independent Experimental Measurements of Diffusion, Sorption, and Permeability Support the Solution‐Diffusion Model of Membrane Transport,” Journal of Membrane Science 737 (2026): 124731, 10.1016/j.memsci.2025.124731.

[79] J. S. Vrentas and J. L. Duda , “Diffusion in Polymer–Solvent Systems. II. A Predictive Theory for the Dependence of Diffusion Coefficients on Temperature, Concentration, and Molecular Weight,” Journal of Polymer Science: Polymer Physics Edition 15 (1977): 417–439, 10.1002/pol.1977.180150303.

[80] J. M. Zielinski and J. L. Duda , “Predicting Polymer/Solvent Diffusion Coefficients Using Free‐Volume Theory,” AIChE Journal 38 (1992): 405–415, 10.1002/AIC.690380309.

[81] M. L. Jewell , B. P. Bengtson , K. Smither , G. Nuti , and T. Perry , “Physical Properties of Silicone Gel Breast Implants,” Aesthetic Surgery Journal 39 (2019): 264–275, 10.1093/ASJ/SJY103. PMC6376345 29718087

[82] F. R. Lourenço , I. S. Kikuchi , T. De , and J. A. Pinto , “Silicone Gel Bleed on Breast Implants,” Surgery 7 (2011): 13.

[83] J. S. Vrentas and C. M. Vrentas , “Solvent Self‐Diffusion in Glassy Polymer‐Solvent Systems,” Macromolecules 27 (1994): 5570–5576, 10.1021/ma00098a009.

[84] J. Crank and G. S. Park , eds., Diffusion in Polymers (n.d.) (Heriot‐Watt University, 2026), https://discovery.hw.ac.uk/primo‐explore/fulldisplay?vid=44HWA_V1&search_scope=HW&tab=jsearch_slot&docid=44hwa_alma2131251340003206&lang=en_US&context=L.

[85] H. R. Moyer , B. H. Ghazi , and A. Losken , “The Effect of Silicone Gel Bleed on Capsular Contracture,” Plastic & Reconstructive Surgery 130 (2012): 793–800, 10.1097/PRS.0B013E318262F174. 22691845

[86] H. B. P. M. Dijkman , I. Slaats , and P. Bult , “Assessment of Silicone Particle Migration Among Women Undergoing Removal or Revision of Silicone Breast Implants in the Netherlands,” JAMA Network Open 4 (2021): 2125381, 10.1001/jamanetworkopen.2021.25381. PMC8453317 34542618

[87] S. Bondurant , V. L. Ernster , and R. Herdman , in Safety of Silicone Breast Implants (National Academies Press, 1999). 20669503

[88] G. P. Maxwell and A. Gabriel , “Breast Implant Design,” Gland Surgery 6 (2017): 148–153, 10.21037/gs.2016.11.09. PMC5409902 28497018

[89] Y. Bachour , Z. C. M. Heinze , T. S. Dormaar , W. G. van Selms , M. J. P. F. Ritt , and F. B. Niessen , “Poly Implant Prothèse Silicone Breast Implants: Implant Dynamics and Capsular Contracture,” European Journal of Plastic Surgery 41 (2018): 563–570, 10.1007/S00238-018-1427-Y. PMC6153863 30294070

[90] A. U. Daniels , “Silicone Breast Implant Materials,” Swiss Medical Weekly: Official Journal of the Swiss Society of Infectious Diseases, the Swiss Society of Internal Medicine, the Swiss Society of Pneumology 142 (2012): 2930, 10.4414/smw.2012.13614.

[91] E. O. Ogbole , J. Lou , S. Ilias , and V. Desmane , “Influence of Surface‐Treated SiO_2_ on the Transport Behavior of O_2_ and N_2_ Through Polydimethylsiloxane Nanocomposite Membrane,” Separation and Purification Technology 175 (2017): 358–364, 10.1016/j.seppur.2016.11.065.

[92] P. Peng , Y. Lan , and J. Luo , “Modified Silica Incorporating Into PDMS Polymeric Membranes for Bioethanol Selection,” Advances in Polymer Technology 2019 (2019): 1–8, 10.1155/2019/5610282.

[93] A. U. Daniels , “Silicone Breast Implant Materials,” Swiss Medical Weekly 142 (2012): 1–12, 10.4414/smw.2012.13614. 22826101

[94] L. Tortolano , S. Yen‐Nicolaÿ , P. F. Rogliano , et al., “Permeability of Expander Breast Implants: In Vitro and In Vivo Analyses,” Journal of the Mechanical Behavior of Biomedical Materials 78 (2018): 427–432, 10.1016/j.jmbbm.2017.12.001. 29223731

[95] Y. J. Fu , H. Z. Qui , K. S. Liao , et al., “Effect of UV‐Ozone Treatment on Poly(dimethylsiloxane) Membranes: Surface Characterization and Gas Separation Performance,” Langmuir 26 (2010): 4392–4399, 10.1021/la903445x. 20214398

[96] D. A. Markov , E. M. Lillie , S. P. Garbett , and L. J. McCawley , “Variation in Diffusion of Gases Through PDMS Due to Plasma Surface Treatment and Storage Conditions,” Biomedical Microdevices 16 (2014): 91–96, 10.1007/s10544-013-9808-2. PMC3945670 24065585

[97] K. Xiang , G. Huang , J. Zheng , X. Wang , G. X. Li , and J. Huang , “Accelerated Thermal Ageing Studies of Polydimethylsiloxane (PDMS) Rubber,” Journal of Polymer Research 19 (2012): 9869, 10.1007/s10965-012-9869-6.

[98] Z. Brounstein , J. Zhao , D. Geller , N. Gupta , and A. Labouriau , “Long‐Term Thermal Aging of Modified Sylgard 184 Formulations,” Polymers 13 (2021): 3125, 10.3390/polym13183125. PMC8466950 34578026

[99] D. E. Dong , J. D. Andrade , and D. L. Coleman , “Adsorption of Low Density Lipoproteins Onto Selected Biomedical Polymers,” Journal of Biomedical Materials Research 21 (1987): 683–700, 10.1002/JBM.820210602. 3597460

[100] R. Carmen and S. C. Mutha , “Lipid Absorption by Silicone Heart Valve Poppets—In‐Vivo and In‐Vitro Results,” Journal of Biomedical Materials Research 6 (1972): 327–346, 10.1002/jbm.820060504. 5072346

[101] F. Bodin , C. Jung , F. Dieval , et al., “Aging of Retrieved Gel Breast Implants: A Comparison Between Two Product Generations,” Journal of the Mechanical Behavior of Biomedical Materials 46 (2015): 11–22, 10.1016/j.jmbbm.2015.02.008. 25746931

[102] W. P. Adams , J. B. Robinson , and R. J. Rohrich , “Lipid Infiltration as a Possible Biologic Cause of Silicone Gel Breast Implant Aging,” Plastic and Reconstructive Surgery 101 (1998): 64–68, 10.1097/00006534-199801000-00011. 9427917

[103] S. P. Barr , E. W. Hill , and A. Bayat , “Novel Proteomic Assay of Breast Implants Reveals Proteins With Significant Binding Differences: Implications for Surface Coating and Biocompatibility,” Aesthetic Surgery Journal 38 (2018): 962–969, 10.1093/asj/sjy018. 29590306

[104] M. W. Keller , S. R. White , and N. R. Sottos , “Torsion Fatigue Response of Self‐Healing Poly(dimethylsiloxane) Elastomers,” Polymer 49 (2008): 3136–3145, 10.1016/j.polymer.2008.04.041.

[105] L. J. Magill , A. Tanska , M. Keshtgar , A. Mosahebi , and G. Jell , “Mechanical and Surface Chemical Analysis of Retrieved Breast Implants From a Single Centre,” Journal of the Mechanical Behavior of Biomedical Materials 91 (2019): 24–31, 10.1016/j.jmbbm.2018.10.042. 30529983

[106] S. S. Teuber , R. L. Saunders , G. M. Halpern , et al., “Elevated Serum Silicon Levels in Women With Silicone Gel Breast Implants,” Biological Trace Element Research 48 (1995): 121–130, 10.1007/BF02789186. 7662498

[107] J. P. McConnell , T. P. Moyer , D. E. Nixon , et al., “Determination of Silicon in Breast and Capsular Tissue From Patients With Breast Implants Performed by Inductively Coupled Plasma Emission Spectroscopy: Comparison With Tissue Histology,” American Journal of Clinical Pathology 107 (1997): 236–246, 10.1093/ajcp/107.2.236. 9024074

[108] S. J. Lugowski , D. C. Smith , H. Bonek , J. Lugowski , W. Peters , and J. Semple , “Analysis of Silicon in Human Tissues With Special Reference to Silicone Breast Implants,” Journal of Trace Elements in Medicine and Biology 14 (2000): 31–42, 10.1016/S0946-672X(00)80021-8. 10836532

[109] M. J. Morykwas , C. P. Calloway , R. Fernando , D. N. Wichems , and B. T. Jones , “Determination of Silicone in Breast Tissue by Graphite Furnace Continuum Source Atomic Absorption Spectrometry,” Applied Spectroscopy 47, no. 10 (1993): 1577–1579, https://opg.optica.org/abstract.cfm?uri=as‐47‐10‐1577.

[110] L. H. Kidder , V. F. Kalasinsky , J. L. Luke , I. W. Levin , and E. N. Lewis , “Visualization of Silicone Gel in Human Breast Tissue Using New Infrared Imaging Spectroscopy,” Nature Medicine 3 (1997): 235–237, 10.1038/nm0297-235. 9018246

[111] L. van Haasterecht , L. Zada , R. W. Schmidt , et al., “Label‐Free Stimulated Raman Scattering Imaging Reveals Silicone Breast Implant Material in Tissue,” Journal of Biophotonics 13 (2020): 201960197, 10.1002/JBIO.201960197. 32049417

[112] E. de Bakker , L. Zada , R. W. Schmidt , et al., “Baker Grade IV Capsular Contracture is Correlated With an Increased Amount of Silicone Material: An Intrapatient Study,” Plastic and Reconstructive Surgery 152 (2023): 1191–1200, 10.1097/PRS.0000000000010359. PMC10666937 36877628

[113] M. Dziubek , R. Laurent , M. Bonapace‐Potvin , L. Gaboury , and M. A. Danino , “Silicone Particles in Capsules Around Breast Implants: Establishment of a New Pathological Methodology to Assess the Number of Particles Around Breast Implants,” Annales de Chirurgie Plastique Esthétique 68 (2023): 19–25, 10.1016/j.anplas.2022.08.002. 36050201

[114] S. Azahaf , K. A. Spit , C. J. M. de Blok , P. Bult , and P. W. B. Nanayakkara , “Silicone Migration From Intact Saline Breast Implants,” Plastic and Reconstructive Surgery—Global Open 12 (2024): 5608, 10.1097/GOX.0000000000005608. PMC10852369 38333026

[115] R. W. Schmidt , E. de Bakker , and F. Ariese , “Silicone Loss During Histological Preparation of Breast Implant Tissue From Capsular Contracture, Quantified by Stimulated Raman Scattering Microscopy,” Journal of Biophotonics 18 (2025): 202400415, 10.1002/jbio.202400415. PMC11793946 39716402

[116] K. Y. Chumbimuni‐Torres , R. E. Coronado , A. M. Mfuh , et al., “Adsorption of Proteins to Thin‐Films of PDMS and Its Effect on the Adhesion of Human Endothelial Cells,” RSC Advances 1 (2011): 706, 10.1039/c1ra00198a. PMC4110918 25068038

[117] X. Zhou , H. Hao , Y. Chen , et al., “Covalently Grafted Human Serum Albumin Coating Mitigates the Foreign Body Response Against Silicone Implants in Mice,” Bioactive Materials 34 (2024): 482–493, 10.1016/j.bioactmat.2024.01.006. PMC10827492 38292409

[118] A. B. Anderson and C. R. Robertson , “Absorption Spectra Indicate Conformational Alteration of Myoglobin Adsorbed on Polydimethylsiloxane,” Biophysical Journal 68 (1995): 2091–2097, 10.1016/S0006-3495(95)80388-7. PMC1282113 7612852

[119] M. Prokopowicz , B. Banecki , J. Łukasiak , and A. Przyjazny , “The Measurement of Conformational Stability of Proteins Adsorbed on Siloxanes,” Journal of Biomaterials Science, Polymer Edition 14 (2003): 103–118, 10.1163/156856203321142560. 12661663

[120] M. Prokopowicz , J. Lukasiak , B. Banecki , and A. Przyjazny , “In Vitro Measurement of Conformational Stability of Fibrinogen Adsorbed on Siloxane,” Biomacromolecules 6 (2005): 39–45, 10.1021/BM040041D. 15638502

[121] L. Kadziński , M. Prokopowicz , J. Jakóbkiewicz‐Banecka , M. Gabig‐Cimińska , J. Łukasiak , and B. Banecki , “Effect of Silicone on the Collagen Fibrillogenesis and Stability,” Journal of Pharmaceutical Sciences 104 (2015): 1275–1281, 10.1002/jps.24351. PMC4418381 25589402

[122] L. M. Nayef , M. F. Khan , and M. A. Brook , “Low Molecular Weight Silicones Particularly Facilitate Human Serum Albumin Denaturation,” Colloids and Surfaces B: Biointerfaces 128 (2015): 586–593, 10.1016/j.colsurfb.2015.03.013. 25800359

[123] J. C. Rosa Neto , P. C. Calder , R. Curi , P. Newsholme , J. K. Sethi , and L. S. Silveira , “The Immunometabolic Roles of Various Fatty Acids in Macrophages and Lymphocytes,” International Journal of Molecular Sciences 22 (2021): 8460, 10.3390/IJMS22168460. PMC8395092 34445165

[124] S. Ferreira , A. S. Barros , and M. Marques , “Breast Implant Illness: Symptoms, Outcomes With Explantation and Potential Etiologies—A Systematic Review and Meta‐Analysis,” Aesthetic Plastic Surgery 49 (2025): 6600–6620, 10.1007/s00266-025-05142-x. PMC12738613 40788544

[125] J. C. R. Mustafá , E. F. C. Fleury , and H. B. P. M. Dijkman , “Case Report: Evidence of Migratory Silicone Particles Arising From Cohesive Silicone Breast Implants,” Frontiers in Women's Health 3 (2022): 730276, 10.3389/fgwh.2022.730276. PMC9085291 35547828

[126] A. Visser , M. M. Hussain , and J. A. Kuivenhoven , “The Intracellular Chylomicron Highway: Novel Insights Into Chylomicron Biosynthesis, Trafficking, and Secretion,” Current Opinion in Lipidology 36 (2025): 145–152, 10.1097/MOL.0000000000000983. PMC12052055 40152288

[127] W. E. Katzin , J. A. Centeno , L. J. Feng , M. Kiley , and F. G. Mullick , “Pathology of Lymph Nodes From Patients With Breast Implants,” American Journal of Surgical Pathology 29 (2005): 506–511, 10.1097/01.PAS.0000155145.60670.E4. 15767806

[128] J. O. Okeme , J. M. Parnis , J. Poole , M. L. Diamond , and L. M. Jantunen , “Polydimethylsiloxane‐Air Partition Ratios for Semi‐Volatile Organic Compounds by GC‐Based Measurement and COSMO‐RS Estimation: Rapid Measurements and Accurate Modelling,” Chemosphere 156 (2016): 204–211, 10.1016/J.CHEMOSPHERE.2016.04.094. 27179237

[129] B. Tansel , “Noncovalent Interactions as Key Modulators of PFAS Translocation, Lipid‐Protein Affinity, and Tissue Partitioning,” Journal of Hazardous Materials 501 (2026): 140933, 10.1016/J.JHAZMAT.2025.140933. 41456435

[130] A. M. Fogelman , B. J. Van Lenten , C. Warden , M. E. Haberland , and P. A. Edwards , “Macrophage Lipoprotein Receptors,” Journal of Cell Science 9 (1988): 135–149, 10.1242/JCS.1988.SUPPLEMENT_9.7. 2855802

[131] K. M. Botham , E. H. Moore , C. De Pascale , and F. Bejta , “The Induction of Macrophage Foam Cell Formation by Chylomicron Remnants,” Biochemical Society Transactions 35 (2007): 454–458, 10.1042/BST0350454. 17511626

[132] N. V. Mushenkova , E. E. Bezsonov , V. A. Orekhova , T. V. Popkova , A. V. Starodubova , and A. N. Orekhov , “Recognition of Oxidized Lipids by Macrophages and Its Role in Atherosclerosis Development,” Biomedicines 9 (2021): 915, 10.3390/BIOMEDICINES9080915. PMC8389651 34440119

[133] I. Khan , L. Timsina , R. Chauhan , et al., “Oxylipins in Breast Implant–Associated Systemic Symptoms,” Aesthetic Surgery Journal 44 (2024): NP695–NP710, 10.1093/ASJ/SJAE128. PMC11403815 38857184

[134] W. L. Nash and M. M. Gabriel , “Ex Vivo Analysis of Cholesterol Deposition for Commercially Available Silicone Hydrogel Contact Lenses Using a Fluorometric Enzymatic Assay,” Eye & Contact Lens: Science & Clinical Practice 40 (2014): 277–282, 10.1097/ICL.0000000000000052. 25099866

[135] A. D. Pucker , M. Thangavelu , and J. J. Nichols , “In Vitro Lipid Deposition on Hydrogel and Silicone Hydrogel Contact Lenses,” Investigative Opthalmology & Visual Science 51 (2010): 6334–6340, 10.1167/IOVS.10-5836. 20702829

[136] J. Löfgren and F. Lohmander , “Silicone Breast Implants and Disease—Many Questions Unanswered,” JAMA Network Open 4 (2021): 2128947, 10.1001/JAMANETWORKOPEN.2021.28947. 34542621

[137] M. Kanduč , W. K. Kim , R. Roa , and J. Dzubiella , “How the Shape and Chemistry of Molecular Penetrants Control Responsive Hydrogel Permeability,” ACS Nano 15 (2021): 614–624, 10.1021/ACSNANO.0C06319/ASSET/IMAGES/LARGE/NN0C06319_0004.JPEG. PMC7844830 33382598

[138] A. Guzman‐Aranguez , B. Colligris , and J. Pintor , “Contact Lenses: Promising Devices for Ocular Drug Delivery,” Journal of Ocular Pharmacology and Therapeutics 29 (2013): 189–199, 10.1089/JOP.2012.0212. 23215541

[139] F. Stapleton , S. Stretton , E. Papas , C. Skotnitsky , and D. F. Sweeney , “Silicone Hydrogel Contact Lenses and the Ocular Surface,” Ocular Surface 4 (2006): 24–43, 10.1016/S1542-0124(12)70262-8. 16669523

[140] K. Walsh , L. W. Jones , P. Morgan , E. B. Papas , and A. Sulley , “Topical Review: Twenty‐Five Years of Silicone Hydrogel Soft Contact Lenses,” Optometry and Vision Science 102 (2025): 361–374, 10.1097/OPX.0000000000002267. PMC12184980 40549698

[141] K. Göntér , S. László , V. Tékus , et al., “New Generation Capsaicin‐Diclofenac Containing, Silicon‐Based Transdermal Patch Provides Prolonged Analgesic Effect in Acute and Chronic Pain Models,” European Journal of Pharmaceutical Sciences 207 (2025): 107035, 10.1016/J.EJPS.2025.107035. 39922237

[142] Ö. Wagner , “Development of a New Silicon‐Based Transdermal System. I. Study of Silicone Elastomers and Effect of Liquid Ingredients,” Drug Development and Industrial Pharmacy 24 (1998): 243–252, 10.3109/03639049809085616. 9876581

[143] B. S. Snorradóttir , P. I. Gudnason , F. Thorsteinsson , and M. Másson , “Experimental Design for Optimizing Drug Release From Silicone Elastomer Matrix and Investigation of Transdermal Drug Delivery,” European Journal of Pharmaceutical Sciences 42 (2011): 559–567, 10.1016/J.EJPS.2011.02.011. 21371556

[144] C. P. Goodrich , M. P. Brenner , and K. Ribbeck , “Enhanced Diffusion by Binding to the Crosslinks of a Polymer Gel,” Nature Communications 9, no. 1 (2018): 4348, 10.1038/s41467-018-06851-5. PMC6195553 30341303

[145] I. Rykowska , I. Nowak , and R. Nowak , “Soft Contact Lenses as Drug Delivery Systems: A Review,” Molecules 26 (2021): 5577, 10.3390/MOLECULES26185577. PMC8472272 34577045

